# Supplementary material for: A longitudinal observational study of home-based conversations for detecting early dementia: protocol for the CUBOId TV task
Source: BMJ Open. 2022 Nov 23;12(11):e065033. doi: 10.1136/bmjopen-2022-065033 (PMC9684963; doi:10.1136/bmjopen-2022-065033)
Supplement: Supplementary data [file bmjopen-2022-065033supp001.pdf]

SUPPLEMENTARY MATERIAL

Supplementary Material S1 (overleaf). Hearing questionnaire to be issued to participants

CUBOId HEARING ASSESSMENT QUESTIONNAIRE

NAME: ____

CURRENT AGE: ____

TODAY'S DATE: ____

I WILL BE COMPLETING THIS FORM (please tick only one box):

Myself ☐

With the help of my study partner ☐

Study partner completed form ☐

HEARING AID STATUS: I wear a hearing aid in my (please tick any that apply):

LEFT EAR ☐ RIGHT EAR ☐

We will ask you to answer 24 questions regarding your hearing experience and abilities. We would like both the CUBOId Main Participant and the Study Partner to answer these questions in relation to their own hearing. All questions require rating your hearing experience on a scale. The questions are in 2 sections, with instructions given at the beginning of each section. The first section asks questions about your "auditory quality of life", and the second section asks questions regarding whether using hearing aids provides any benefit for those difficulties when watching TV. The questions in each section are subtly different so please answer all questions in all sections if possible- if an answer does not apply to you (for example, you do not wear hearing aids), please answer "not applicable".

____

SECTION 1

15-item Speech, Spatial and Qualities of Hearing Questionnaire

These 15 questions inquire about aspects of your ability and experience while hearing and listening in different situations. For each question, put a mark, such as a cross, anywhere on the scale shown against each question. For example, putting a mark at 10 means that you would be perfectly able to do or experience what is described in the question. Putting a mark at 0 means you would be quite unable to do or experience what is described. As an example, question 1 asks about having a conversation with someone while the TV is on at the same time. If you are well able to do this, then put a mark toward the right-hand end of the scale. If you could follow about half the conversation in this situation put the mark around the mid-point, and so on. We expect that all the questions are relevant to your everyday experience, but if a question describes a situation that does not apply to you, put a cross in the "not applicable" box. Please also write a note next to that question explaining why it does not apply in your case.

1. **You are talking with one other person and there is a TV on in the same room. Without turning the TV down, can you follow what the person you're talking to says?**

Not at all *Perfectly*

0 1 2 3 4 5 6 7 8 9 10 Not applicable ☐

2. **You are in a group of about 5 people in a busy restaurant. You CAN see everyone else in the group. Can you follow the conversation?**

Not at all *Perfectly*

0 1 2 3 4 5 6 7 8 9 10 Not applicable ☐

3. **You are talking with one other person. There is continuous background noise, such as a fan or running water. Can you follow what the person says?**

Not at all *Perfectly*

0 1 2 3 4 5 6 7 8 9 10 Not applicable ☐

4. **You are in a group of about 5 people in a busy restaurant. You CANNOT see everyone else in the group. Can you follow the conversation?**

Not at all *Perfectly*

0 1 2 3 4 5 6 7 8 9 10 Not applicable ☐

5. **You are in conversation with one person in a room where there are many other people talking. Can you follow what the person you are talking to is saying?**

Not at all *Perfectly*

0 1 2 3 4 5 6 7 8 9 10 Not applicable ☐

6. **You are sitting around a table or at a meeting with several people. You can't see everyone. Can you tell where any person is as soon as they start speaking?**

Not at all *Perfectly*

0 1 2 3 4 5 6 7 8 9 10 Not applicable ☐

7. **You are outside. A dog barks loudly. Can you tell immediately where it is, without having to look?**

Not at all *Perfectly*

0 1 2 3 4 5 6 7 8 9 10 Not applicable ☐

8. **You are standing on the footpath of a busy street. Can you hear right away which direction a bus or truck is coming from before you see it?**

Not at all *Perfectly*

0 1 2 3 4 5 6 7 8 9 10 Not applicable ☐

9. Can you tell from the sound of their voice or footsteps which direction a person is moving, for example, from your left to your right, or right to left?

Not at all — *Perfectly*

0 1 2 3 4 5 6 7 8 9 10

Not applicable ☐

10. Do you have the impression of sounds being exactly where you would expect them to be?

Not at all — *Perfectly*

0 1 2 3 4 5 6 7 8 9 10

Not applicable ☐

11. Do you find it easy to recognise different people you know by the sound of each one's voice?

Not at all — *Perfectly*

0 1 2 3 4 5 6 7 8 9 10

Not applicable ☐

12. Do you find it easy to distinguish different pieces of music that you are familiar with?

Not at all — *Perfectly*

0 1 2 3 4 5 6 7 8 9 10

Not applicable ☐

13. Can you tell the difference between different sounds, for example, a car versus a bus; water boiling in a pot versus food cooking in a frying pan?

Not at all — *Perfectly*

0 1 2 3 4 5 6 7 8 9 10

Not applicable ☐

14. When you listen to music, does it sound clear and natural?

Not at all *Perfectly*

0 1 2 3 4 5 6 7 8 9 10 Not applicable ☐

15. Do everyday sounds that you can hear easily seem clear to you (not blurred)?

Not at all *Perfectly*

0 1 2 3 4 5 6 7 8 9 10 Not applicable ☐

SECTION 2

Glasgow Hearing Aid Benefit Profile

In this section we describe 3 different listening scenarios and ask the same three questions about each of them. Please read all three scenarios before answering them, as they target similar but slightly different listening situations. Please circle the answer that best applies to your listening experience. Please assume that the term "hearing aid(s)" applies whether you wear one or two hearing aid(s). If you do not use hearing aid(s), please only answer the first question in each section and "N/A" for the other two. It would be helpful if you could also write a note saying "no HAs", so we can be sure that is why the question wasn't answered.

1. **You are listening to the TV with other family or friends when the volume is adjusted to suit other people:**

a. What level of difficulty do you have in this situation without hearing aid(s)?

N/A None Slight Moderate Great Cannot manage

b. In this situation, how much do your hearing aid(s) help you?

N/A Not at all A little Moderately Quite a lot A great deal

c. In this situation, with your hearing aid(s), how much difficulty do you now have?

N/A None A little Moderate Quite a lot A great deal

2. You are following TV when watching alone when there is no background noise:

a. What level of difficulty do you have in this situation without hearing aid(s)?

N/A None Slight Moderate Great Cannot manage

b. In this situation, how much do your hearing aid(s) help you?

N/A Not at all A little Moderately Quite a lot A great deal

c. In this situation, with your hearing aid(s), how much difficulty do you now have?

N/A None A little Moderate Quite a lot A great deal

3. You are listening to someone talking to you while at the same time listening to a newscaster on TV. You are trying to follow what both people are saying.

a. What level of difficulty do you have in this situation without hearing aid(s)?

N/A None Slight Moderate Great Cannot manage

b. In this situation, how much do your hearing aid(s) help you?

N/A Not at all A little Moderately Quite a lot A great deal

c. In this situation, with your hearing aid(s), how much difficulty do you now have?

N/A None A little Moderate Quite a lot A great deal

Supplementary Material S2. Test battery recommended in the European Prevention of Alzheimer's Dementia (EPAD) study. Table adapted from [51].

Cognitive Domain	Test	Platform
Reaction time/information processing speed/conceptual shifting/selective attention	Coding	RBANS
Verbal episodic memory	List learning	RBANS
	Story memory	RBANS
Visuospatial analysis	Figure copy	RBANS
	Line orientation	RBANS
Language	Picture naming	RBANS
	Semantic fluency	RBANS
Working memory	Digit span	RBANS
	Dot counting	NIH examiner/toolbox
Allocentric space	Four mountains task	iPad app
Paired associate learning	Paired associate learning	NIH examiner/toolbox
