# Supplementary material for: A longitudinal observational study of home-based conversations for detecting early dementia: protocol for the CUBOId TV task
Source: BMJ Open. 2022 Nov 23;12(11):e065033. doi: 10.1136/bmjopen-2022-065033 (PMC9684963; doi:10.1136/bmjopen-2022-065033)
Supplement: Author's manuscript [file bmjopen-2022-065033.draft_revisions.pdf]

BMJ Open

BMJ Open is committed to open peer review. As part of this commitment we make the peer review history of every article we publish publicly available.

When an article is published we post the peer reviewers' comments and the authors' responses online. We also post the versions of the paper that were used during peer review. These are the versions that the peer review comments apply to.

The versions of the paper that follow are the versions that were submitted during the peer review process. They are not the versions of record or the final published versions. They should not be cited or distributed as the published version of this manuscript.

BMJ Open is an open access journal and the full, final, typeset and author-corrected version of record of the manuscript is available on our site with no access controls, subscription charges or pay-per-view fees (http://bmjopen.bmj.com).

If you have any questions on BMJ Open's open peer review process please email info.bmjopen@bmj.com

BMJ Open

The use of home-based conversations for detecting early dementia: Protocol for the CUBOId TV task

Journal:	*BMJ Open*
Manuscript ID	bmjopen-2022-065033
Article Type:	Protocol
Date Submitted by the Author:	21-May-2022
Complete List of Authors:	Kumpik, Daniel; University of Bristol, Department of Engineering Mathematics<br>Santos-Rodriguez, Raul; University of Bristol, Department of Engineering Mathematics<br>Selwood, James; Bristol Medical School; University of Bristol, Department of Population Health Sciences<br>Coulthard , Liz ; Bristol Medical School; University of Bristol, Department of Translational Health Sciences<br>Twomey, Niall; University of Bristol, Department of Electrical & Electronic Engineering<br>Craddock, Ian ; University of Bristol, Department of Electrical and Electronic Engineering<br>Ben-Shlomo, Yoav; University of Bristol, Department of Population Health Sciences
Keywords:	Dementia < NEUROLOGY, Health informatics < BIOTECHNOLOGY & BIOINFORMATICS, GERIATRIC MEDICINE, Neurology < INTERNAL MEDICINE, Adult neurology < NEUROLOGY, Neuropathology < NEUROLOGY

SCHOLARONE™
Manuscripts

The use of home-based conversations for detecting early dementia: Protocol for the CUBOId TV task

Authors

Daniel Paul Kumpik[1], Raul Santos-Rodriguez[1], James Selwood[2], Elizabeth Coulthard[3], Niall Twomey[4], Ian Craddock[4], Yoav Ben-Shlomo[2].

1. Department of Engineering Mathematics, University of Bristol, Bristol, UK

2. Population Health Sciences, Bristol Medical School, University of Bristol, Bristol, UK

3. Translational Health Sciences, Bristol Medical School, University of Bristol, Bristol, UK

4. Department of Electrical & Electronic Engineering, University of Bristol, Bristol, UK

Author for correspondence: Daniel Kumpik, Department of Engineering Mathematics, Ada Lovelace Building, Tankard's Close, University Walk, Bristol BS8 1TW; dan.kumpik@bristol.ac.uk

Word count: 3998

For peer review only

ABSTRACT

Introduction

Limitations in effective dementia therapies mean that early diagnosis and monitoring are critical for disease management, but current clinical tools are impractical and/or unreliable, and disregard short-term symptom variability. Behavioural biomarkers of cognitive decline, such as speech, sleep and activity patterns, can manifest prodromal pathological changes. They can be continuously measured at home with smart sensing technologies, and permit leveraging of interpersonal interactions for optimising diagnostic and prognostic performance. Here we describe the ContinUous behavioural Biomarkers Of cognitive Impairment (CUBOId) study, which explores the feasibility of multimodal data fusion for in-home monitoring of mild cognitive impairment (MCI) and early Alzheimer's disease (AD). The report focuses on a subset of CUBOId participants who perform a novel speech task, the "TV task", designed to track changes in ecologically valid conversations with disease progression.

Methods and Analysis

Participants have diagnoses of MCI or AD, and controls are their live-in partners with no such diagnosis. Multimodal activity data were passively acquired from wearables and in-home fixed sensors over periods ranging from 8 to 25 months. At 2 time points participants completed the TV task over 5 days by recording audio of their conversations as they watched a favourite TV programme, with further testing to be completed after removal of the sensor installations. Behavioural testing is supported by neuropsychological assessment for deriving behavioural ground truths on cognitive status. Deep learning will be used to generate fused multimodal activity-speech embeddings for optimisation of diagnostic and predictive performance from speech alone.

Ethics and dissemination

CUBOId was approved by an NHS Research Ethics Committee (Wales REC; ref: 18/WA/0158) and is sponsored by University of Bristol. It is also supported by the National Institute for Health Research (NIHR) Clinical Research Network West of England. Results will be reported at conferences and in peer-reviewed scientific journals.

Keywords

dementia, mild cognitive impairment, Alzheimer's disease, behavioural biomarkers, activities of daily living, speech, smart sensing technologies, multimodal data fusion, deep learning

For peer review only - http://bmjopen.bmj.com/site/about/guidelines.xhtml

ARTICLE SUMMARY

Strengths and limitations

- To our knowledge, this is the first study to simultaneously characterise longitudinal, ecologically valid diagnostic trajectories across a broad range of behavioural domains relevant to cognitive decline "in the wild", allowing construction of shared multimodal embeddings for dementia diagnosis from speech alone
- Participants' live-in partners are contextually matched controls, accounting for differences across participants' home environments and lifestyles
- Measurements of cognitive status from neuropsychological testing form ground truths for behavioural biomarkers of cognitive decline
- Limited sample size and restricted demographics may confer limited generalisability of findings to other populations
- Behavioural and neuropsychological testing timelines disrupted and desynchronised due to COVID-19

INTRODUCTION

Alzheimer's disease (AD) is one of the most common neurological diseases, affecting an estimated 42 million people worldwide [1]. AD causes a progressive loss of memory, executive, visuospatial and linguistic functions, which disrupts patients' ability to engage in functional activities of daily living (ADL) [2]. Therapeutic options are limited, and clinical interventions are likely more effective in very early or prodromal stages of AD, highlighting the value of early detection [3]. However, abnormal brain pathology can manifest decades before cognitive symptoms [4] and by the time of diagnosis 50% of patients are beyond the early stages [5].

Mild cognitive impairment (MCI), often a prodromal phase of AD, is characterised by forgetfulness, mood changes, and navigation, decision-making and word-finding problems, which are not sufficiently severe to interfere with ADL [6]. Up to 50% of individuals diagnosed with MCI develop AD within 5 years [7], but some MCI cases remain stable and some recover over time [8], complicating prediction of an individual patient's disease progression. Clinical investigations such as lumbar puncture and brain imaging are expensive and impractical for large-scale deployment [9,10], so clinicians have traditionally relied on cognitive screening tests [11]. However, these tests may be limited in validity, specificity, inter-examiner consistency, disregard short-term symptom variability,

For peer review only - http://bmjopen.bmj.com/site/about/guidelines.xhtml

and usually require stressful visits to clinic, which can bias results [12,13]. They therefore provide a contextually sparse and relatively insensitive measure of subtle early signs of cognitive decline [4,13]. Whilst blood biomarkers are close to clinical implementation and could revolutionise detection of molecular AD pathology [14], these are positive early in the course of the illness, so do not mirror clinical decline, and can yield false positives.

These limitations have driven recent interest in passive technologies for longitudinal monitoring of behavioural dementia biomarkers in naturalistic settings [15–17]. Preclinical disruptions in activities such as speaking [18], environmental navigation [19], sleep [20] and walking [21] can betray distal biomarkers such as wandering, circadian disturbances, apathy and agitation [22], which may be useful both in diagnosing MCI and predicting which patients with MCI may go on to develop AD. These ecologically valid, everyday behaviours can be measured continuously and unobtrusively in the home, using networked fixed sensors, or portable sensors in wearables, smartphones and tablets [16]. Fusion across sensing modalities permits fine-grained contextual analysis of how engagement in different ADL covaries over days, weeks and months, and automated machine learning (ML) techniques can leverage intraindividual variability reflective of early cognitive changes [15,23]. These technologies have potential to help clinicians build personalised and holistic digital phenotypes for prediction of neurodegeneration [17,24]. However, they remain insufficiently mature for full-scale clinical deployment [15].

Speech is a particularly interesting dementia biomarker because linguistic competence whilst cognitively healthy is a known predictor for developing AD [25,26]. AD disrupts morphological, lexical, semantic, syntactic and structural features of language [27], and can even be detected in acoustic, temporal and other paralinguistic features [28–30]. Speech is simple to acquire using modern smart devices and therefore is an attractive target for longitudinal in-home monitoring of cognitive decline [31]. Advances in automated speech recognition (ASR), natural language processing (NLP) and other ML technologies have contributed to promising results in classifying AD from recorded speech [32], and recent work has also shown that linguistic and paralinguistic features can help detect MCI [33]. However, classification remains more challenging for MCI than AD, probably because MCI patients' cognitive profiles are heterogenous and not as far removed from those of normal ageing [28,34].

Studies leveraging speech to diagnose dementia often train ML models on databases of structured speech gleaned using narrative tasks such as picture description. These provide a rich impression of semantic content, idea density, syntactic complexity, speech fluency and paralinguistic parameters, and discourse fluency [35], which could be important features for detecting prodromal AD. However,

For peer review only - http://bmjopen.bmj.com/site/about/guidelines.xhtml

other than occasional interventions from a clinical investigator (which are often discarded (e.g., [36]), narrative monologues are constrained by the stimulus material and impoverished in verbal interactions found in natural conversations. Spontaneous conversations require executive attentional, metacognitive and linguistic dexterities not demanded by structured speech tasks that may be useful for detecting MCI due to AD or predicting cognitive decline [35,37]. Moreover, picture description tasks mostly preclude dyadic signs of cognitive decline, such as prompting in response to word-finding problems [38]. Indeed, the importance of dyadic features for dementia diagnosis is emphasised by the recent finding that it is possible to classify AD from picture description tasks with an accuracy of 78% from the speech of the interviewer alone [39]. Whilst there is acknowledgement that leveraging ecologically valid, naturalistic conversations for diagnosing dementia has merit [40,41], speech is rarely collected via passive, *unprompted* observation of patients and their partners “in the wild”, prompting recent calls for investigation of ecologically acquired, conversational speech relevant to ADL that could confer better diagnostic generalisability [42].

The ContinUous behavioural Biomarkers Of cognitive Impairment (CUBOId) study at the University of Bristol (UoB) is part of the EPSRC Sensor Platform for HEalthcare in a Residential Environment (SPHERE) Interdisciplinary Research Collaboration [43]. CUBOId aims to advance the state of the art in multimodal in-home sensing for novel dementia-related behavioural biomarkers [44] such as sleep disturbances, partner shadowing, wandering and disrupted conversational speech. We will develop novel computational algorithms to infer biomarkers of cognitive decline as measured using sensor networks in MCI and AD patients’ own homes, permitting autonomous, continuous, long-term behavioural sensing. Where possible these will be validated against neuroimaging (amyloid PET) data and neuropsychological progression over time. The patient’s live-in companions are also participants, to act as environmentally matched controls and shed light on interactional biomarkers of cognitive decline. Because the companion is familiar to the patient, we hope to record behavioural interactions that have high ecological validity.

Our objectives are as follows:

1. To develop ML models capable of leveraging home-acquired, natural speech for automated prediction, diagnosis and longitudinal tracking of cognitive decline. For this we will establish ground truths on early speech biomarkers of MCI/AD from neurological and neuropsychological tests.
2. To leverage our multimodal dataset to investigate how temporal variability in ADL behaviours is reflected in clinically relevant variability in linguistic proficiency. For example,

For peer review only - http://bmjopen.bmj.com/site/about/guidelines.xhtml

we hypothesise that periods of agitation, wandering and sleep disturbances will precede disturbed speech as revealed by acoustic and prosodic changes, hesitations, mnemonic search markers and endogenous (e.g., rapid speech) and exogenous (e.g., calming conversational interactions) linguistic features.

3. To use data fusion techniques to leverage our multi-modal data streams by deriving latent relational embeddings between ADL and speech for optimisation of diagnostic and predictive performance from speech alone.

Whilst CUBOId's sensing regime is intended to be comprehensive, we aim to define the minimum dataset required for disease detection and monitoring, to enable more cost-efficient future use with simpler data capture methods. A further outcome will be pilot data on effect sizes sufficient to generate a meaningful power calculation to guide future studies. Here we report on the data acquisition and analysis protocol for CUBOId's novel conversational speech task, the "TV task".

METHODS

This is a longitudinal observational "proof of concept" study to examine speech and other behavioural biomarkers amongst cases with MCI or early AD and their partners, who act as controls. Speech is repeatedly measured over time in parallel with repeat neuropsychology and continuous multimodal home sensing. This permits investigation of whether speech-based diagnosis and monitoring of early dementia can be optimised using simultaneously recorded behavioural data. CUBOId started on 22/03/2019 and runs until 31/12/2022.

Patient and public involvement (PPI)

Prior to CUBOId deployments, SPHERE ran co-design workshops and publicly trialled its consent processes. On average, 86% properly understood the risks and 92% consented to take part. Patients were involved in pilot experiments for CUBOId's behavioural tasks. A PPI advisory team gave recommendations on recruitment methods, materials provided to participants and other practical issues. We plan to involve the PPI team with dissemination of materials to participants, the public and healthcare professionals.

Participants and eligibility criteria

For peer review only - http://bmjopen.bmj.com/site/about/guidelines.xhtml

For peer review only

Although data collection is ongoing, recruitment has now ended. Participants were recruited via the cognitive neurology service at the North Bristol NHS Trust Brain Centre, the Bristol Dementia Wellbeing Service and the Research Institute for the Care of Older People in Bath. Informed consent was obtained by a clinical fellow (JS) at the North Bristol Trust Brain Centre.

Participant pairs, each consisting of an outpatient with a previous diagnosis of either MCI or AD (confirmed by JS & EC) and their partner or live-in carer with no such diagnosis, were eligible. Participants with MCI were offered MRI and amyloid PET brain scans on a voluntary basis to discern the likely aetiology of their cognitive complaint (e.g. AD, vascular disease, subjective cognitive disorder). Where available, these were complemented with routine cerebrospinal fluid measurements of amyloid-β 42 and total tau expressed as total tau/amyloid-β 42 ratio, using a validated cut-off used in clinical practice above which an AD diagnosis is thought likely.

Participants with severe comorbidities were not considered. Participants were selected according to the following criteria; (a) Full capacity to give informed consent; (b) At least 50 years old; (c) Competent English speaker; (d) Normal or corrected-to-normal vision; (e) Consent to have SPHERE installed in their home.

A conventional sample size calculation was not performed as CUBOId is a precursor to future larger scale evaluations if the results look hopeful. From the larger CUBOId participant pool 9 sets of participants are participating in the TV task, of which 8 are patient-companion pairs with one MCI or AD patient (4/8 female). All 8 participant pairs have one male and one female participant. An additional male MCI patient lives alone, but nevertheless verbally describes the TV show during each recording session, permitting insight into how TV task performance differs under monadic and dyadic conditions. The mean age (± SD) of MCI and AD patients at study entry was 71.4 ± 6.9 years, and the mean age of companions was 64 ± 14 years (this information was not available for one participant pair). Companions are usually the life partners of the patients (one case is an adult child who lives with their parent). 6/9 patients had prior MCI diagnoses and 3/9 had prior AD diagnoses.

Some participants have hearing problems, a known risk factor for cognitive decline. We will therefore issue participants with a custom hearing questionnaire derived from the Speech, Spatial and Qualities of Hearing questionnaire (short form) [45] and the Glasgow Hearing Aid Benefit Profile [46] (see supplemental material S1).

The novel paradigm of the TV task

For peer review only - http://bmjopen.bmj.com/site/about/guidelines.xhtml

This task was inspired by the TV show “Gogglebox”. Participants record their conversations as they watch a TV programme of their choice such as the news, soap operas or other regular shows. They do so for a minimum of 30 minutes, at roughly the same time of day, over 5 consecutive days. It is emphasised that these are idealised guidelines and that participants should prioritise daily activities and unforeseen events over the TV task recording schedule. Participants are therefore asked, at minimum, to provide 5 half-hour recordings regardless of spacing between recordings. To catch discrepancies in TV shows or timings of recordings across days, participants recite the date and time and the name of the show at the beginning of each recording.

Participants place the tablet between their normal sitting positions for watching TV, then activate the audio recording app and converse naturally whilst using the TV programme as a stimulus for conversation. This guideline is to emphasise our desire to acquire naturalistic conversation, but also to maximise the amount of speech we record. Participants are asked to leave the app recording during adverts and if possible, until the end of the show. Once participants acquire 5 half-hour recordings, they return the tablet to the CUBOId team via courier.

Speech recordings are made using a Samsung Galaxy SM-T580 tablet and a pre-installed app [1], chosen because it is free of advertisements and easy to use, whilst providing a good range of recording options. Audio files are recorded at a sample rate of 44.1 kHz in mono in WAV format, apart from one pilot data collection block, which was recorded in MP3 format.

Tablets were provided by the research team during home visits prior to COVID-19’s arrival, but are now couriered to participants. Couriered tablets, chargers and parcel tape for return packaging are disinfected with antiviral spray, and a minimum three-day period is enforced after packing before participants open the package. Then, at a pre-agreed time, participants are contacted by the CUBOId team and given training on the TV task either over the phone or securely using Zoom video conferencing software [47].

Participants are shown how to use the Audio Recorder app and how to delete a recording if necessary for privacy reasons. After this, companions and patients provide controlled speech recordings by reading aloud ten sentences from the Harvard list [48], a set of phonetically balanced English sentences used to assess speech intelligibility in telecommunications systems. These control recordings provide a template for training deep neural networks to diarise participants’ speech.

[1] https://github.com/Dimowner/AudioRecorder

For peer review only - http://bmjopen.bmj.com/site/about/guidelines.xhtml

Participants then perform the TV task at their convenience. Detailed instructions describing the recording procedure are included as a PDF file on the device home screen, and telephone support is available in case of unforeseen difficulties.

Impact of COVID-19 on TV task data collection timeline

COVID-19, and technician/researcher availability, have caused severe disruptions to CUBOId by desynchronising the behavioural tasks. We originally intended to collect behavioural data across 3-4 testing blocks per participant pair, held at roughly 0, 6 and 12 months after SPHERE installation, with the possibility of a fourth block at 18 months if possible. We intended to visit participants' homes to train them on the behavioural tasks, but we suspended SPHERE deployments in March 2020 and introduced remote testing protocols to mitigate risk to participants. One participant pair first performed the TV task prior to the pandemic and had a large gap (17 months) between the first and second TV task administrations. The remaining participant sets completed the first TV task block in January-March 2021, after which one set withdrew from the TV task. All remaining participant sets completed their second block in July-September 2021, six of which still concurrently had active SPHERE deployments installed. A third testing block will be completed before 31st December 2022, without sensors present in participants' homes. To date, 9 participant sets have completed TV task testing block 1, 8 have completed block 2, and block 3 is currently underway.

Neuropsychological and cognitive tasks

Participants completed baseline neuropsychological testing (administered by JS: see supplemental material S2) before SPHERE installation. We intended to conduct repeat assessments at 6 and 12 months follow-up, but COVID-19 restrictions delayed these until later in the study. The test battery was adapted from the recommendations of the European Prevention of Alzheimer's Dementia (EPAD) study [49]. Participants also completed the Alzheimer's Disease Assessment Scale – Cognitive subscale (ADAS-Cog), considered the gold standard for dementia research trials [50].

Participants' psychological wellbeing and sleep were assessed using the 6-Item State-Trait Anxiety Inventory [51], the Geriatric Depression Scale (Short Version) [52], the Epworth Sleepiness Scale [53] and the Pittsburgh Sleep Quality Index [54]. After COVID-19's arrival, neuropsychological and cognitive assessments were conducted using the online video-conferencing platform Attend Anywhere [2].

[2] https://www.attendanywhere.com

For peer review only - http://bmjopen.bmj.com/site/about/guidelines.xhtml

To measure memory performance participants complete a memory task at home using the Mezurio smartphone app [55] after each TV task testing block. Participants are encouraged to play Mezurio at roughly the same time each day, for about 10 minutes per day, for 30 days.

Multimodal sensing with SPHERE

SPHERE sensor modalities are categorised as wearable, environmental or silhouette. Custom wrist-worn wearables record acceleration indicative of bodily activity, posture and movement, and room-level location via triangulation of received signal strength measurements at Bluetooth access points throughout the home. The environmental modality consists of room sensors for occupancy, temperature, humidity, water and electricity usage. Silhouette cameras yield whole-body pose as well as fine-grained location information.

DATA ANALYSIS

We will use Python and associated libraries for all analyses unless otherwise stated. Features derived from SPHERE sensors and speech recordings will be selected by examining their contributions to the variance in each dataset, their correlations with clinical measures such as ADAS-Cog and from insights into how ADL and speech covary longitudinally with disease progression.

Location and activity detection from SPHERE sensor data

Dedicated pre-processing pipelines will be applied for different sensor types, but fusion across sensors will provide a spatially and temporally contextualised overview of ADL behaviours and anomalies. We will explore which sensors are best combined for different activities and at what level of integration, and also the optimal stages at which to fuse activity with location data, ranging from early (naïve multimodal feature set integration) to late (hierarchical integration of posterior probabilities from deep learning models trained on individual feature sets) fusion strategies [23,56].

Activity detection and in-home localization will leverage the sequential nature of our data by recognizing that certain behavioural transitions follow others with characteristic probabilities (Figure 1). For example, people never transition directly from sitting to running, but often from sitting to standing- and then running. Similarly, changes in participant location can only proceed across adjacent positions in physical space. This sequential dependence of states can be modelled using Viterbi decoding, which tracks a maximum-likelihood path through a series of noisy, probabilistic transitions in a Hidden Markov Model (HMM) [56,57]. We will compare HMM activity classification

For peer review only - http://bmjopen.bmj.com/site/about/guidelines.xhtml

and localization performance to deep learning architectures capable of leveraging spatial and temporal relationships in the multimodal feature space such as convolutional neural networks and long short-term memory networks [23]. We will compare these with pipelines exploiting both activity and location data together for further disambiguation of behaviour.

At the highest analysis level, we will derive sensor feature combinations suggestive of periods of "abnormal" dementia-related behaviours. For example, wandering could be inferred from a joint comparison of the wearable's activity levels and location complexity [44].

Behaviours we have identified as likely to be indicative of cognitive decline include [3]:

- Wandering, and shadowing of the companion
- Sleep disturbances
- Agitation
- Apathy, sedentarisation

We will analyse these longitudinally to identify short periods of unusual behaviour and longer-term changes that may be indicative of disease progression- and anomalous events in the recent past or future that could be related to such changes, such as poor sleep during nights before an episode of agitation. To leverage these behaviours alongside our conversational speech data, we will use representation learning to generate novel latent activity/speech embeddings for dementia detection based on speech alone. We will project these novel features onto unseen speech samples after exploring different strategies for fusing SPHERE sensor data with our paralinguistic and linguistic models.

Speech pre-processing

We will build deep learning pipelines comprising paralinguistic and NLP domains to detect MCI, and predict the probability of progression to AD, from conversational speech, after experimenting with low and high-level fusion strategies for paralinguistic and linguistic features. The speech recordings suffer from quality issues that will require a dedicated pre-processing and speaker diarisation pipeline. Firstly, the samples contain two participants who speak infrequently (and sometimes over each other) against a variable TV background. The TV produces structured sounds including music, speech and sound effects with unpredictable spectrotemporal statistics that often resemble the participants' voices (Figure 2). Our single-channel recording setup is challenging for standard source separation techniques, but because we have acquired controlled voice recordings from each participant, we will use a semi-supervised learning architecture (such as variational autoencoders) to

For peer review only - http://bmjopen.bmj.com/site/about/guidelines.xhtml

For peer review only

derive latent features for target separation. PRAAT phonetics software [58] will be used for annotating participants' voices for evaluation purposes, and recordings will be transcribed for NLP modelling according to established protocols [59].

Acoustic and paralinguistic features

We will extract non-linguistic speech features at local (i.e., fast Fourier transform window duration), semi-local (i.e., central tendency and variability statistics of features calculated on time scales of seconds) and global (i.e., over an entire audio file) time scales. Guided by feature taxonomies in a recent systematic review [60], local features will include representations of the speech spectrogram, such as Mel-frequency cepstral coefficients, as well as spectral bandwidth, short-time energy and zero-crossing count. Semi-local features will comprise paralinguistic measures of vocal quality known to reflect neuropathology such as jitter and shimmer, and higher prosodic features such as intonation, stress and rhythm. At the global feature level, we will compute utterance statistics such as articulation rate, time spent talking and mean utterance duration, and temporal mnemonic features relating to hesitations. We will extract interactional and pragmatic features such as the number of turn changes and overlapping segments [61], number of repair sequences [62] and inter-turn silence duration [63].

Natural language processing

We will analyse transcripts of CUBOId participants' speech using NLP modelling and deep learning. Python's Natural Language Toolkit and similar open resources will be used to conduct analyses on cognition-relevant features at the lexical, connected language and semantic levels [27]. At the lexical level, tokenisation and stemming, morphological parsing and part-of-speech tagging will help derive measures of lexical diversity. At the level of connected language, we will explore pragmatics, syntactic complexity and correctness, topic coherence, and sequence predictability measures of disordered thinking. At the semantic level we will characterise idea density, a measure of linguistic conceptual richness highly predictive of cognitive decline in later life, and we will experiment with word embeddings selected from pre-trained language models such as BERT [64], which have found a cutting-edge role in dementia detection studies.

ETHICS AND DISSEMINATION

A major issue for studies with vulnerable people is to ensure they understand and are willing participants. Therefore JS, a clinician with extensive experience of working with dementia patients,

For peer review only - http://bmjopen.bmj.com/site/about/guidelines.xhtml

ensured that all participants had capacity to consent at the outset. Given CUBOId's longitudinal nature, it is possible that capacity may be lost over time. Should this occur the partner is asked their opinion as to whether or not it is appropriate to proceed based on the patient's previously stated preferences.

CUBOId is sponsored by UoB and was approved by the Wales Research Ethics Committee (REC) 7 (ref: 18/WA/0158) and the Health Research Authority (IRAS project ID 234027). CUBOId is supported by the National Institute for Health Research (NIHR) Clinical Research Network West of England. Protocol amendments and adverse events are referred to the REC and UoB. UoB has Public Liability and Professional Negligence insurance policies to cover the eventuality of harm to a research participant or University employee arising from design or management of the research. Unforeseen protocol deviations are reported to the Principal Investigator, and serious deviations to the Sponsor, immediately. If CUBOId is ended prematurely, the Principal Investigator will notify the UoB and Wales RECs, including the reasons for termination.S

DATA STATEMENT

All individuals in the CUBOId team have unrestricted access to the full dataset. Results will be reported at conferences, in peer-reviewed scientific journals and on the UoB website.

Both the anonymised data and study documentation are stored on secure servers within UoB and shared according to UoB procedures and guidelines. Personally identifiable information is stored separately to research data. Data management procedures follow the stipulations of the General Data Protection Regulation. In line with NIHR guidance, we obtained consent for participants' data to be stored for up to 20 years and shared anonymously with other researchers, after appropriate review to ensure it will be used according to the ethics approval.

SUMMARY

To our knowledge there is no full-scale deployment of in-home, real-world, longitudinal activity sensing technologies that is complemented by monitoring of naturalistic conversations and neuropsychological assessment of MCI or AD patients. By bringing together these behavioural domains, CUBOId will observe how they covary with disease progression, permitting construction of models that perform better than those trained on a single modality. Here, we present a novel

For peer review only - http://bmjopen.bmj.com/site/about/guidelines.xhtml

protocol for recording speech from MCI and AD patients and their companions as they converse at home. We intend to develop, test and de-risk a novel speech-based diagnostic asset for the UK Dementias Research Institute community, which may be applied to cohorts in the Dementia Platform UK, and the international community.

Author contributions: YBS, EC, JS, RSR, NT & IC conceived and designed CUBOId. DK, YBS, EC, JS, RSR & NT designed the TV task, and DK collected the TV task data and drafted the manuscript. All authors reviewed the manuscript prior to submission.

Acknowledgements: We would like to thank Julia Carey, David Bailey and Danielle Hale for their support in administering CUBOId and in collecting data. We would also like to thank our participants for their involvement in CUBOId.

Competing interests: None declared.

Funding: This research was funded by CUBOId (UK MRC Momentum grant MC/PC/16029), the SPHERE IRC (grant EP/K031910/1) and supported by the EPSRC Digital Health and Care Centre for Doctoral Training (CDT) at the University of Bristol (UKRI Grant No. EP/S023704/1).

REFERENCES

1 Brookmeyer R, Johnson E, Ziegler-Graham K, *et al.* Forecasting the global burden of Alzheimer's disease. *Alzheimers Dement* 2007;**3**:186–91. doi:10.1016/J.JALZ.2007.04.381

2 Saari T, Hallikainen I, Hintsa T, *et al.* Neuropsychiatric symptoms and activities of daily living in Alzheimer's disease: ALSOVA 5-year follow-up study. *Int Psychogeriatr* 2020;**32**:741–51. doi:10.1017/S1041610219001571

3 Liberati G, Raffone A, Belardinelli MO. Cognitive reserve and its implications for rehabilitation and Alzheimer's disease. *Cogn Process* 2012;**13**:1–12. doi:10.1007/s10339-011-0410-3

4 Bateman RJ, Fagan AM, Holtzman DM, *et al.* Clinical and biomarker changes in dominantly inherited Alzheimer' s disease. *N Engl J Med* 2012;**367**:795–804. doi:10.1056/NEJMoa1202753

5 Hodge S, Hailey E. Second English National Memory Clinics Audit Report. London: Royal College of Psychiatrists, 2015.

6 Langa KM, Levine DA. The Diagnosis and Management of Mild Cognitive Impairment: A Clinical Review. *JAMA* 2014;**312**:2551. doi:10.1001/JAMA.2014.13806

For peer review only - http://bmjopen.bmj.com/site/about/guidelines.xhtml

7 Petersen RC. Mild cognitive impairment as a diagnostic entity. *J Intern Med* 2004;**256**:183–94. doi: 10.1111/j.1365-2796.2004.01388.x

8 Ganguli M, Dodge HH, Shen C, *et al.* Mild cognitive impairment, amnestic type: an epidemiologic study. *Neurology* 2004;**63**:115–21. doi:10.1212/01.WNL.0000132523.27540.81

9 Ruan Q, D'Onofrio G, Sancarlo D, *et al.* Potential neuroimaging biomarkers of pathologic brain changes in Mild Cognitive Impairment and Alzheimer's disease: A systematic review. *BMC Geriatr* 2016;**16**:1–9. doi:10.1186/S12877-016-0281-7

10 Mo JA, Lim JH, Sul AR, *et al.* Cerebrospinal Fluid β-Amyloid1–42 Levels in the Differential Diagnosis of Alzheimer's Disease—Systematic Review and Meta-Analysis. *PLoS One* 2015;**10**:e0116802. doi:10.1371/JOURNAL.PONE.0116802

11 Webster L, Groskreutz D, Grinbergs-Saull A, *et al.* Development of a core outcome set for disease modification trials in mild to moderate dementia: a systematic review, patient and public consultation and consensus recommendations. *Health Technol Assess (Winchester, England)* 2017;**21**. doi:10.3310/HTA21260

12 Tsoi KKF, Chan JYC, Hirai HW, *et al.* Cognitive Tests to Detect Dementia: A Systematic Review and Meta-analysis. *JAMA Intern Med* 2015;**175**:1450–8. doi:10.1001/JAMAINTERNMED.2015.2152

13 Brown J. The use and misuse of short cognitive tests in the diagnosis of dementia. *J Neurol Neurosurg Psychiatry* 2015;86:680–5. doi:10.1136/jnnp-2014-309086

14 Teunissen CE, Verberk IMW, Thijssen EH, *et al.* Blood-based biomarkers for Alzheimer's disease: towards clinical implementation. *Lancet Neurol* 2022;**21**:66–77. doi:10.1016/S1474-4422(21)00361-6

15 Piau A, Wild K, Mattek N, *et al.* Current state of digital biomarker technologies for real-life, home-based monitoring of cognitive function for mild cognitive impairment to mild Alzheimer disease and implications for clinical care: Systematic review. *J Med Internet Res* 2019;**21**. doi:10.2196/12785

16 Gillani N, Arslan T. Intelligent Sensing Technologies for the Diagnosis, Monitoring and Therapy of Alzheimer's Disease: A Systematic Review. *Sensors* 2021;**21**:4249. doi:10.3390/S21124249

17 Palliya Guruge C, Oviatt S, Delir Haghighi P, *et al.* Advances in Multimodal Behavioral Analytics for Early Dementia Diagnosis: A Review. *Proceedings of the 2021 International Conference on Multimodal Interaction*;**13**. doi:10.1145/3462244

18 Drummond C, Coutinho G, Fonseca RP, *et al.* Deficits in narrative discourse elicited by visual stimuli are already present in patients with mild cognitive impairment. *Front Aging Neurosci* 2015;**7**:96. doi:10.3389/FNAGI.2015.00096

19 Howett D, Castegnaro A, Krzywicka K, *et al.* Differentiation of mild cognitive impairment using an entorhinal cortex-based test of virtual reality navigation. *Brain* 2019;**142**:1751–66. doi:10.1093/brain/awz116

20 Gagnon JF, Lafrenière A, Rauchs G, *et al.* Sleep in Normal Aging, Alzheimer's Disease, and Mild Cognitive Impairment. In: Nomikos G, Feltner D, eds. Handbook of Behavioural Neuroscience: Translational Medicine in CNS Drug Development. Elsevier, 2019:677–92. doi:10.1016/B978-0-12-813743-7.00045-1

For peer review only - http://bmjopen.bmj.com/site/about/guidelines.xhtml

21 Masse FAA, Ansai JH, Fiogbe E, *et al.* Progression of Gait Changes in Older Adults with Mild Cognitive Impairment: A Systematic Review. *J Geriatr Phys Ther* 2021;**44**:119–24. doi:10.1519/JPT.0000000000000281

22 Kales HC, Gitlin LN, Lyketsos CG. Assessment and management of behavioral and psychological symptoms of dementia. *BMJ* 2015;**350**. doi:10.1136/bmj.h369

23 Qiu S, Zhao H, Jiang N, *et al.* Multi-sensor information fusion based on machine learning for real applications in human activity recognition: State-of-the-art and research challenges. *Inf Fusion* 2022;**80**:241–65. doi:10.1016/J.INFFUS.2021.11.006

24 Kourtis LC, Regele OB, Wright JM, *et al.* Digital biomarkers for Alzheimer's disease: the mobile/wearable devices opportunity. *NPJ Digit Med* 2019;**2**:1–9. doi:10.1038/s41746-019-0084-2

25 Iacono D, Markesbery WR, Gross M, *et al.* The Nun Study: Clinically silent AD, neuronal hypertrophy, and linguistic skills in early life. *Neurology* 2009;**73**:665–73. doi:10.1212/WNL.0b013e3181b01077

26 Eyigoz E, Mathur S, Santamaria M, *et al.* Linguistic markers predict onset of Alzheimer's disease. *EClinicalMedicine* 2020;**28**:100583. doi:10.1016/j.eclinm.2020.100583

27 Clarke N, Foltz P, Garrard P. How to do things with (thousands of) words: Computational approaches to discourse analysis in Alzheimer's disease. *Cortex* 2020;**129**:446–63. doi:10.1016/J.CORTEX.2020.05.001

28 Gosztolya G, Vincze V, Tóth L, *et al.* Identifying Mild Cognitive Impairment and mild Alzheimer's disease based on spontaneous speech using ASR and linguistic features. *Comput Speech Lang* 2019;**53**:181–97. doi:10.1016/j.csl.2018.07.007

29 Haider F, de La Fuente S, Luz S. An Assessment of Paralinguistic Acoustic Features for Detection of Alzheimer's Dementia in Spontaneous Speech. *IEEE J Sel Top Signal Process* 2020;**14**:272–81. doi:10.1109/JSTSP.2019.2955022

30 Gonzalez-Moreira E, Torres-Boza D, Garcia-Zamora MA, *et al.* Prosodic speech analysis to identify mild cognitive impairment. *IFMBE Proc* 2015;**49**:580–3. doi:10.1007/978-3-319-13117-7_148

31 Fraser KC, Komeili M. Measuring Cognitive Status from Speech in a Smart Home Environment. *IEEE Instrum Meas Mag* 2021;**24**:13–21. doi:10.1109/MIM.2021.9513645

32 Petti U, Baker S, Korhonen A. A systematic literature review of automatic Alzheimer's disease detection from speech and language. *J Am Med Inform Assoc* 2020;**27**,1784–1797. doi:10.1093/jamia/ocaa174

33 Martínez-Nicolás I, Llorente TE, Martínez-Sánchez F, *et al.* Ten Years of Research on Automatic Voice and Speech Analysis of People With Alzheimer's Disease and Mild Cognitive Impairment: A Systematic Review Article. *Front Psychol* 2021;**12**:645. doi:10.3389/FPSYG.2021.620251

34 König A, Satt A, Sorin A, *et al.* Automatic speech analysis for the assessment of patients with predementia and Alzheimer's disease. *Alzheimers Dement* 2015;**1**:112–24. doi:10.1016/j.dadm.2014.11.012

For peer review only - http://bmjopen.bmj.com/site/about/guidelines.xhtml

35 Mueller KD, Hermann B, Mecollari J, *et al.* Connected speech and language in mild cognitive impairment and Alzheimer's disease: A review of picture description tasks. *J Clin Exp Neuropsychol* 2018;**40**:917–39. doi:10.1080/13803395.2018.1446513

36 Shah Z, Sawalha J, Tasnim M, *et al.* Learning Language and Acoustic Models for Identifying Alzheimer's Dementia From Speech. *Front Comput Sci* 2021;**0**:4. doi:10.3389/FCOMP.2021.624659

37 Asgari M, Kaye J, Dodge H. Predicting mild cognitive impairment from spontaneous spoken utterances. *Alzheimers Dement* 2017;**3**:219. doi:10.1016/J.TRCI.2017.01.006

38 Nasreen S, Hough J, Purver M. Rare-Class Dialogue Act Tagging for Alzheimer's Disease Diagnosis. In *Proceedings of the 22nd Annual Meeting of the Special Interest Group on Discourse and Dialogue* 2021;290-300.

39 Pérez-Toro PA, Bayerl SP, Arias-Vergara T, *et al.* Influence of the Interviewer on the Automatic Assessment of Alzheimer's Disease in the Context of the ADReSSo Challenge. In *Proceedings of Interspeech 2021* 2021; 3785–3789. doi:10.21437/Interspeech.2021-1589

40 Gkoumas D, Wang B, Tsakalidis A, *et al.* A Longitudinal Multi-modal Dataset for Dementia Monitoring and Diagnosis. *arXiv* 2109.01537 [Preprint]. September 03, 2021 [cited 2022 Mar 28] https://doi.org/10.48550/arXiv.2109.01537

41 Davis BH, Pope C. Finding a balance: The Carolinas Conversation Collection. *Corpus Linguist Linguist Theory* 2011;**7**:143–61. doi:10.1515/CLLT.2011.007

42 de La Fuente Garcia S, Ritchie C, Luz S. Artificial Intelligence, speech and language processing approaches to monitoring Alzheimer's Disease: a systematic review. *J Alzheimers Dis* 2020;**78**:1547-1574.

43 Woznowski P, Burrows A, Diethe T, *et al.* SPHERE: A Sensor Platform for Healthcare in a Residential Environment. In: Angelakis V, Tragos E, Pöhls H, Kapovtis A, Bassi A, eds. Designing, Developing, and Facilitating Smart Cities. Springer, 2017:315–33. doi:10.1007/978-3-319-44924-1_14

44 Poyiadzi R, Yang W, Ben-Shlomo Y, *et al.* Detecting Signatures of Early-stage Dementia with Behavioural Models Derived from Sensor Data. *CEUR Workshop Proc* 2020;**2820**:23–7.

45 Moulin A, Vergne J, Gallego S, *et al.* A New Speech, Spatial, and Qualities of Hearing Scale Short-Form: Factor, Cluster, and Comparative Analyses. *Ear hear* 2019;**40**:938–50. doi:10.1097/AUD.0000000000000675

46 Gatehouse, S. The Glasgow Hearing Aid Benefit Profile: What it measures and how to use it. *Hear J* 2000;53:10–18.

47 Zoom Video Communications Inc. Zoom Security White Paper. Retrieved 28 March 2022 from https://explore.zoom.us/docs/doc/Zoom-Security-White-Paper.pdf.

48 Williams CE, Hecker MHL. Relation between Intelligibility Scores for Four Test Methods and Three Types of Speech Distortion. *J Acoust Soc Am* 2005;**44**:1002. doi:10.1121/1.1911189

49 Ritchie K, Ropacki M, Albala B, *et al.* Recommended cognitive outcomes in preclinical Alzheimer's disease: Consensus statement from the European Prevention of Alzheimer's Dementia project. *Alzheimers Dement* 2017;**13**:186–95. doi:10.1016/J.JALZ.2016.07.154

For peer review only - http://bmjopen.bmj.com/site/about/guidelines.xhtml

50 Kueper JK, Speechley M, Montero-Odasso M. The Alzheimer's Disease Assessment Scale–Cognitive Subscale (ADAS-Cog): Modifications and Responsiveness in Pre-Dementia Populations. A Narrative Review. *J Alzheimers Dis* 2018;**63**:423–44. doi:10.3233/JAD-170991

51 Marteau TM, Bekker H. The development of a six-item short-form of the state scale of the Spielberger State—Trait Anxiety Inventory (STAI). *British J Clin Psychol* 1992;**31**:301–6. doi:10.1111/J.2044-8260.1992.TB00997.X

52 Sheikh JI, Yesavage JA. Geriatric Depression Scale (GDS): Recent evidence and development of a shorter version. *Clin Gerontol* 1986;**5**:165–73. doi:10.1300/J018V05N01_09

53 Johns MW. A New Method for Measuring Daytime Sleepiness: The Epworth Sleepiness Scale. *Sleep* 1991;**14**:540–5. doi:10.1093/SLEEP/14.6.540

54 Buysse DJ, Reynolds CF, Monk TH, *et al.* The Pittsburgh sleep quality index: A new instrument for psychiatric practice and research. *Psychiatry Res* 1989;**28**:193–213. doi:10.1016/0165-1781(89)90047-4

55 Lancaster C, Koychev I, Blane J, *et al.* Evaluating the Feasibility of Frequent Cognitive Assessment Using the Mezurio Smartphone App: Observational and Interview Study in Adults With Elevated Dementia Risk. *JMIR Mhealth Uhealth* 2020;8:e16142. doi:10.2196/16142

56 Kozlowski M, Byrne D, Santos-Rodriguez R, *et al.* Data fusion for robust indoor localisation in digital health. *2018 IEEE Wireless Communications and Networking Conference Workshops, WCNCW 2018* 2018;:302–7. doi:10.1109/WCNCW.2018.8369009

57 Lentzas A, Vrakas D. Non-intrusive human activity recognition and abnormal behavior detection on elderly people: a review. *Artif Intell Rev* 2020;**53**:1975–2021. doi:10.1007/S10462-019-09724-5

58 Boersma P, Weenink D. Praat: doing phonetics by computer [Computer program]. Version 6.2.10, retrieved 28 March 2022 from http://www.praat.org/.

59 Macwhinney B. Tools for Analyzing Talk Part 1: The CHAT Transcription Format. Carnegie, 2022. Retrieved 28 March 2022 from https://talkbank.org/manuals/CHAT.pdf. https://doi.org/10.21415/3mhn-0z89

60 Voleti R, Liss JM, Berisha V. A Review of Automated Speech and Language Features for Assessment of Cognitive and Thought Disorders. *IEEE J Sel Top Signal Process* 2020;**14**:282–98. doi:10.1109/JSTSP.2019.2952087

61 Young JA, Lind C, van Steenbrugge W. A conversation analytic study of patterns of overlapping talk in conversations between individuals with dementia and their frequent communication partners. *Int J Lang Commun Disord* 2016;**51**:745–56. doi:10.1111/1460-6984.12245

62 Samuelsson C, Hydén LC. Collaboration, trouble and repair in multiparty interactions involving couples with dementia or aphasia. *Int J Speech Lang Pathol* 2017;**19**:454–464. doi:10.1080/17549507.2016.1221448

63 Nasreen S, Rohanian M, Hough J, *et al.* Alzheimer's Dementia Recognition From Spontaneous Speech Using Disfluency and Interactional Features. *Front Comput Sci* 2021;**3**:1–15. doi:10.3389/fcomp.2021.640669

For peer review only

64 Roshanzamir A, Aghajan H, Soleymani Baghshah M. Transformer-based deep neural network language models for Alzheimer's disease risk assessment from targeted speech. *BMC Med Inform Decis Mak* 2021;**21**:1–14. doi:10.1186/S12911-021-01456-3

For peer review only

FIGURE LEGENDS

Figure 1. Hidden Markov Models for activity detection. Activity transitions can be modelled as state changes in a Markov chain, with hidden activity states x_{ti} at time *t* represented by observed sensor (e.g., wearable accelerometer) inputs z_{ti}. Activities are assumed to transition sequentially with fixed probabilities obtained empirically (shown by the thickness and shading of the grey arrows): in this example, walking generally transitions to standing more often than to sitting, and never directly to lying down or cycling because a standing transition is required first. Maximum likelihood estimation over the entire sequence of sensor inputs is used to obtain an optimal activity transition sequence.

Figure 2. TV task speech pre-processing for speaker diarisation. *Top*, raw speech waveform with overlapping speakers 1 and 2 labelled, as well as the TV. *Middle*, speech spectrogram with zero-crossing count, a feature useful for detecting voiced and unvoiced speech, overlaid. *Bottom*, silence removal using a naïve energy detector after careful denoising.

For peer review only

For peer review only - http://bmjopen.bmj.com/site/about/guidelines.xhtml

Licence Statement

I Daniel Paul Kumpik, the Submitting Author has the right to grant and does grant on behalf of all authors of the Work (as defined in the below author licence), an exclusive licence and/or a non-exclusive licence for contributions from authors who are: i) UK Crown employees; ii) where BMJ has agreed a CC-BY licence shall apply, and/or iii) in accordance with the terms applicable for US Federal Government officers or employees acting as part of their official duties; on a worldwide, perpetual, irrevocable, royalty-free basis to BMJ Publishing Group Ltd ("BMJ") its licensees and where the relevant Journal is co-owned by BMJ to the co-owners of the Journal, to publish the Work in BMJ Open and any other BMJ products and to exploit all rights, as set out in our licence.

The Submitting Author accepts and understands that any supply made under these terms is made by BMJ to the Submitting Author unless you are acting as an employee on behalf of your employer or a postgraduate student of an affiliated institution which is paying any applicable article publishing charge ("APC") for Open Access articles. Where the Submitting Author wishes to make the Work available on an Open Access basis (and intends to pay the relevant APC), the terms of reuse of such Open Access shall be governed by a Creative Commons licence – details of these licences and which Creative Commons licence will apply to this Work are set out in our licence referred to above.

For peer review only - http://bmjopen.bmj.com/site/about/guidelines.xhtml

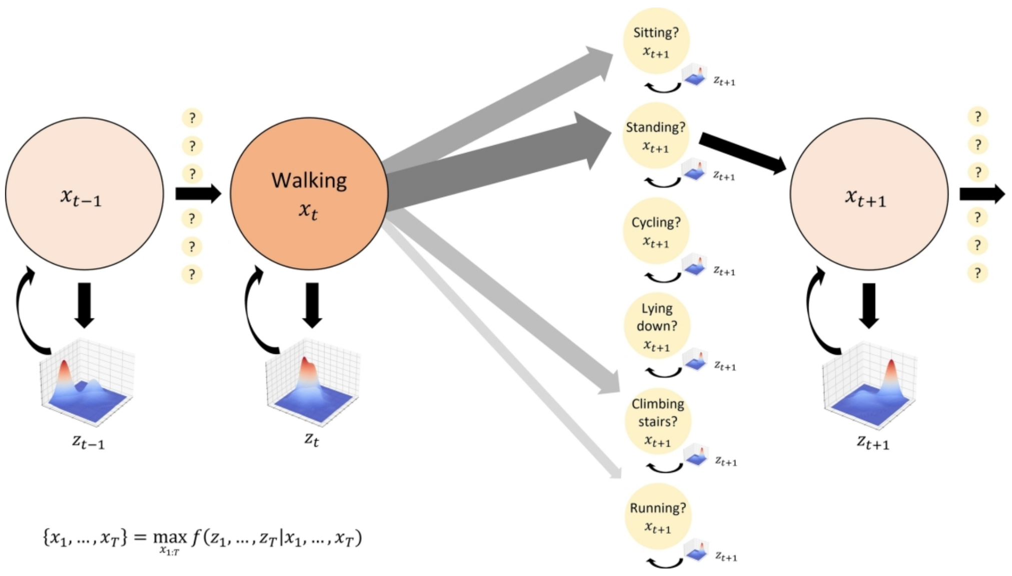

Figure 1. Hidden Markov Models for activity detection. Activity transitions can be modelled as state changes in a Markov chain, with hidden activity states x_ti at time t represented by observed sensor (e.g., wearable accelerometer) inputs z_ti. Activities are assumed to transition sequentially with fixed probabilities obtained empirically (shown by the thickness and shading of the grey arrows): in this example, walking generally transitions to standing more often than to sitting, and never directly to lying down or cycling because a standing transition is required first. Maximum likelihood estimation over the entire sequence of sensor inputs is used to obtain an optimal activity transition sequence.

91x51mm (600 x 600 DPI)

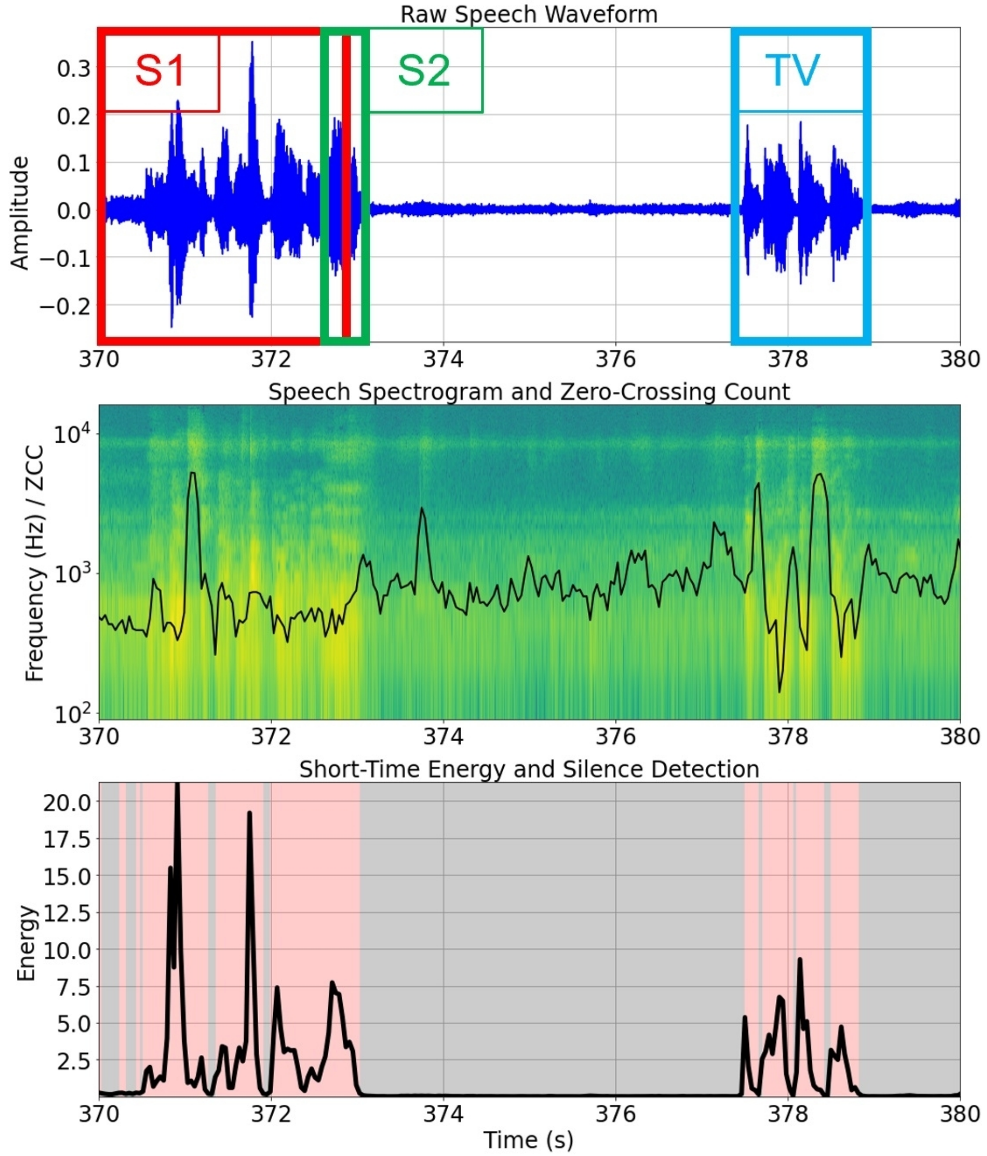

Figure 2. TV task speech pre-processing for speaker diarisation. Top, raw speech waveform with overlapping speakers 1 and 2 labelled, as well as the TV. Middle, speech spectrogram with zero-crossing count, a feature useful for detecting voiced and unvoiced speech, overlaid. Bottom, silence removal using a naïve energy detector after careful denoising.

96x112mm (300 x 300 DPI)

SUPPLEMENTAL MATERIAL

S1 (overleaf). Hearing questionnaire to be issued to participants

For peer review only

CUBOId HEARING ASSESSMENT QUESTIONNAIRE

NAME: ______________________________

CURRENT AGE: ______________________________

TODAY'S DATE: ______________________________

I WILL BE COMPLETING THIS FORM (please tick only one box):

Myself ☐

With the help of my study partner ☐

Study partner completed form ☐

HEARING AID STATUS: I wear a hearing aid in my (please tick any that apply):

LEFT EAR ☐ RIGHT EAR ☐

We will ask you to answer 24 questions regarding your hearing experience and abilities. We would like both the CUBOId Main Participant and the Study Partner to answer these questions in relation to their own hearing. All questions require rating your hearing experience on a scale. The questions are in 2 sections, with instructions given at the beginning of each section. The first section asks questions about your "auditory quality of life", and the second section asks questions regarding whether using hearing aids provides any benefit for those difficulties when watching TV. The questions in each section are subtly different so please answer all questions in all sections if possible- if an answer does not apply to you (for example, you do not wear hearing aids), please answer "not applicable".

For peer review only

SECTION 1

15-item Speech, Spatial and Qualities of Hearing Questionnaire

These 15 questions inquire about aspects of your ability and experience while hearing and listening in different situations. For each question, put a mark, such as a cross, anywhere on the scale shown against each question. For example, putting a mark at 10 means that you would be perfectly able to do or experience what is described in the question. Putting a mark at 0 means you would be quite unable to do or experience what is described. As an example, question 1 asks about having a conversation with someone while the TV is on at the same time. If you are well able to do this, then put a mark toward the right-hand end of the scale. If you could follow about half the conversation in this situation put the mark around the mid-point, and so on. We expect that all the questions are relevant to your everyday experience, but if a question describes a situation that does not apply to you, put a cross in the "not applicable" box. Please also write a note next to that question explaining why it does not apply in your case.

1. **You are talking with one other person and there is a TV on in the same room. Without turning the TV down, can you follow what the person you're talking to says?**

Not at all ... *Perfectly*

0 1 2 3 4 5 6 7 8 9 10

Not applicable ☐

2. **You are in a group of about 5 people in a busy restaurant. You CAN see everyone else in the group. Can you follow the conversation?**

Not at all ... *Perfectly*

0 1 2 3 4 5 6 7 8 9 10

Not applicable ☐

3. **You are talking with one other person. There is continuous background noise, such as a fan or running water. Can you follow what the person says?**

Not at all ... *Perfectly*

0 1 2 3 4 5 6 7 8 9 10

Not applicable ☐

For peer review only - http://bmjopen.bmj.com/site/about/guidelines.xhtml

4. You are in a group of about 5 people in a busy restaurant. You CANNOT see everyone else in the group. Can you follow the conversation?

Not at all *Perfectly*

0 1 2 3 4 5 6 7 8 9 10

Not applicable ☐

5. You are in conversation with one person in a room where there are many other people talking. Can you follow what the person you are talking to is saying?

Not at all *Perfectly*

0 1 2 3 4 5 6 7 8 9 10

Not applicable ☐

6. You are sitting around a table or at a meeting with several people. You can't see everyone. Can you tell where any person is as soon as they start speaking?

Not at all *Perfectly*

0 1 2 3 4 5 6 7 8 9 10

Not applicable ☐

7. You are outside. A dog barks loudly. Can you tell immediately where it is, without having to look?

Not at all *Perfectly*

0 1 2 3 4 5 6 7 8 9 10

Not applicable ☐

8. You are standing on the footpath of a busy street. Can you hear right away which direction a bus or truck is coming from before you see it?

Not at all *Perfectly*

0 1 2 3 4 5 6 7 8 9 10

Not applicable ☐

9. Can you tell from the sound of their voice or footsteps which direction a person is moving, for example, from your left to your right, or right to left?

Not at all *Perfectly*

0 1 2 3 4 5 6 7 8 9 10

Not applicable ☐

10. Do you have the impression of sounds being exactly where you would expect them to be?

Not at all *Perfectly*

0 1 2 3 4 5 6 7 8 9 10

Not applicable ☐

11. Do you find it easy to recognise different people you know by the sound of each one's voice?

Not at all *Perfectly*

0 1 2 3 4 5 6 7 8 9 10

Not applicable ☐

12. Do you find it easy to distinguish different pieces of music that you are familiar with?

Not at all *Perfectly*

0 1 2 3 4 5 6 7 8 9 10

Not applicable ☐

13. Can you tell the difference between different sounds, for example, a car versus a bus; water boiling in a pot versus food cooking in a frying pan?

Not at all *Perfectly*

0 1 2 3 4 5 6 7 8 9 10

Not applicable ☐

14. When you listen to music, does it sound clear and natural?

Not at all *Perfectly*

0 1 2 3 4 5 6 7 8 9 10 Not applicable ☐

15. Do everyday sounds that you can hear easily seem clear to you (not blurred)?

Not at all *Perfectly*

0 1 2 3 4 5 6 7 8 9 10 Not applicable ☐

SECTION 2

Glasgow Hearing Aid Benefit Profile

In this section we describe 3 different listening scenarios and ask the same three questions about each of them. Please read all three scenarios before answering them, as they target similar but slightly different listening situations. Please circle the answer that best applies to your listening experience. Please assume that the term “hearing aid(s)” applies whether you wear one or two hearing aid(s). If you do not use hearing aid(s), please only answer the first question in each section and “N/A” for the other two. It would be helpful if you could also write a note saying “no HAs”, so we can be sure that is why the question wasn’t answered.

1. **You are listening to the TV with other family or friends when the volume is adjusted to suit other people:**

a. What level of difficulty do you have in this situation without hearing aid(s)?

N/A None Slight Moderate Great Cannot manage

b. In this situation, how much do your hearing aid(s) help you?

For peer review only - http://bmjopen.bmj.com/site/about/guidelines.xhtml

N/A Not at all A little Moderately Quite a lot A great deal

c. In this situation, with your hearing aid(s), how much difficulty do you now have?

N/A None A little Moderate Quite a lot A great deal

2. You are following TV when watching alone when there is no background noise:

a. What level of difficulty do you have in this situation without hearing aid(s)?

N/A None Slight Moderate Great Cannot manage

b. In this situation, how much do your hearing aid(s) help you?

N/A Not at all A little Moderately Quite a lot A great deal

c. In this situation, with your hearing aid(s), how much difficulty do you now have?

N/A None A little Moderate Quite a lot A great deal

3. You are listening to someone talking to you while at the same time listening to a newscaster on TV. You are trying to follow what both people are saying.

a. What level of difficulty do you have in this situation without hearing aid(s)?

N/A None Slight Moderate Great Cannot manage

b. In this situation, how much do your hearing aid(s) help you?

N/A Not at all A little Moderately Quite a lot A great deal

c. In this situation, with your hearing aid(s), how much difficulty do you now have?

N/A None A little Moderate Quite a lot A great deal

For peer review only

S2. Test battery recommended in the European Prevention of Alzheimer's Dementia (EPAD) study. Table adapted from [49].

Cognitive Domain	Test	Platform
Reaction time/information processing speed/conceptual shifting/selective attention	Coding	RBANS
Verbal episodic memory	List learning	RBANS
	Story memory	RBANS
Visuospatial analysis	Figure copy	RBANS
	Line orientation	RBANS
Language	Picture naming	RBANS
	Semantic fluency	RBANS
Working memory	Digit span	RBANS
	Dot counting	NIH examiner/toolbox
Allocentric space	Four mountains task	iPad app
Paired associate learning	Paired associate learning	NIH examiner/toolbox

For peer review only

STANDARD PROTOCOL ITEMS: RECOMMENDATIONS FOR INTERVENTIONAL TRIALS

SPIRIT 2013 Checklist: Recommended items to address in a clinical trial protocol and related documents*

Section/item	Item No	Description	Addressed on page number
**Administrative information**			
Title	1	Descriptive title identifying the study design, population, interventions, and, if applicable, trial acronym	_______1______
Trial registration	2a	Trial identifier and registry name. If not yet registered, name of intended registry	______________
	2b	All items from the World Health Organization Trial Registration Data Set	______________
Protocol version	3	Date and version identifier	page headers
Funding	4	Sources and types of financial, material, and other support	_______14_____
Roles and responsibilities	5a	Names, affiliations, and roles of protocol contributors	_______1, 14___
	5b	Name and contact information for the trial sponsor	_______13_____
	5c	Role of study sponsor and funders, if any, in study design; collection, management, analysis, and interpretation of data; writing of the report; and the decision to submit the report for publication, including whether they will have ultimate authority over any of these activities	______________
	5d	Composition, roles, and responsibilities of the coordinating centre, steering committee, endpoint adjudication committee, data management team, and other individuals or groups overseeing the trial, if applicable (see Item 21a for data monitoring committee)	______________

For peer review only - http://bmjopen.bmj.com/site/about/guidelines.xhtml

Introduction

Background and rationale	6a	Description of research question and justification for undertaking the trial, including summary of relevant studies (published and unpublished) examining benefits and harms for each intervention	3-5
	6b	Explanation for choice of comparators	4-5
Objectives	7	Specific objectives or hypotheses	5-6
Trial design	8	Description of trial design including type of trial (eg, parallel group, crossover, factorial, single group), allocation ratio, and framework (eg, superiority, equivalence, noninferiority, exploratory)	6

Methods: Participants, interventions, and outcomes

Study setting	9	Description of study settings (eg, community clinic, academic hospital) and list of countries where data will be collected. Reference to where list of study sites can be obtained	7-8
Eligibility criteria	10	Inclusion and exclusion criteria for participants. If applicable, eligibility criteria for study centres and individuals who will perform the interventions (eg, surgeons, psychotherapists)	7
Interventions	11a	Interventions for each group with sufficient detail to allow replication, including how and when they will be administered	7-9
	11b	Criteria for discontinuing or modifying allocated interventions for a given trial participant (eg, drug dose change in response to harms, participant request, or improving/worsening disease)	12-13
	11c	Strategies to improve adherence to intervention protocols, and any procedures for monitoring adherence (eg, drug tablet return, laboratory tests)	8,9
	11d	Relevant concomitant care and interventions that are permitted or prohibited during the trial	
Outcomes	12	Primary, secondary, and other outcomes, including the specific measurement variable (eg, systolic blood pressure), analysis metric (eg, change from baseline, final value, time to event), method of aggregation (eg, median, proportion), and time point for each outcome. Explanation of the clinical relevance of chosen efficacy and harm outcomes is strongly recommended	5
Participant timeline	13	Time schedule of enrolment, interventions (including any run-ins and washouts), assessments, and visits for participants. A schematic diagram is highly recommended (see Figure)	9

For peer review only - http://bmjopen.bmj.com/site/about/guidelines.xhtml

Sample size	14	Estimated number of participants needed to achieve study objectives and how it was determined, including clinical and statistical assumptions supporting any sample size calculations	_____7_______
Recruitment	15	Strategies for achieving adequate participant enrolment to reach target sample size	_____________

Methods: Assignment of interventions (for controlled trials)

Allocation:

Sequence generation	16a	Method of generating the allocation sequence (eg, computer-generated random numbers), and list of any factors for stratification. To reduce predictability of a random sequence, details of any planned restriction (eg, blocking) should be provided in a separate document that is unavailable to those who enrol participants or assign interventions	_____________
Allocation concealment mechanism	16b	Mechanism of implementing the allocation sequence (eg, central telephone; sequentially numbered, opaque, sealed envelopes), describing any steps to conceal the sequence until interventions are assigned	_____________
Implementation	16c	Who will generate the allocation sequence, who will enrol participants, and who will assign participants to interventions	_____________
Blinding (masking)	17a	Who will be blinded after assignment to interventions (eg, trial participants, care providers, outcome assessors, data analysts), and how	_____________
	17b	If blinded, circumstances under which unblinding is permissible, and procedure for revealing a participant's allocated intervention during the trial	_____________

Methods: Data collection, management, and analysis

Data collection methods	18a	Plans for assessment and collection of outcome, baseline, and other trial data, including any related processes to promote data quality (eg, duplicate measurements, training of assessors) and a description of study instruments (eg, questionnaires, laboratory tests) along with their reliability and validity, if known. Reference to where data collection forms can be found, if not in the protocol	____7-10_____
	18b	Plans to promote participant retention and complete follow-up, including list of any outcome data to be collected for participants who discontinue or deviate from intervention protocols	______9______

Data management	19	Plans for data entry, coding, security, and storage, including any related processes to promote data quality (eg, double data entry; range checks for data values). Reference to where details of data management procedures can be found, if not in the protocol	______13_____
Statistical methods	20a	Statistical methods for analysing primary and secondary outcomes. Reference to where other details of the statistical analysis plan can be found, if not in the protocol	_____10-12____
	20b	Methods for any additional analyses (eg, subgroup and adjusted analyses)	____________
	20c	Definition of analysis population relating to protocol non-adherence (eg, as randomised analysis), and any statistical methods to handle missing data (eg, multiple imputation)	____________
**Methods: Monitoring**			
Data monitoring	21a	Composition of data monitoring committee (DMC); summary of its role and reporting structure; statement of whether it is independent from the sponsor and competing interests; and reference to where further details about its charter can be found, if not in the protocol. Alternatively, an explanation of why a DMC is not needed	____________
	21b	Description of any interim analyses and stopping guidelines, including who will have access to these interim results and make the final decision to terminate the trial	____________
Harms	22	Plans for collecting, assessing, reporting, and managing solicited and spontaneously reported adverse events and other unintended effects of trial interventions or trial conduct	_____13______
Auditing	23	Frequency and procedures for auditing trial conduct, if any, and whether the process will be independent from investigators and the sponsor	____________
**Ethics and dissemination**			
Research ethics approval	24	Plans for seeking research ethics committee/institutional review board (REC/IRB) approval	_____13______
Protocol amendments	25	Plans for communicating important protocol modifications (eg, changes to eligibility criteria, outcomes, analyses) to relevant parties (eg, investigators, REC/IRBs, trial participants, trial registries, journals, regulators)	_____13______

For peer review only - http://bmjopen.bmj.com/site/about/guidelines.xhtml

Consent or assent	26a	Who will obtain informed consent or assent from potential trial participants or authorised surrogates, and how (see Item 32)	____12-13____
	26b	Additional consent provisions for collection and use of participant data and biological specimens in ancillary studies, if applicable	_____13______
Confidentiality	27	How personal information about potential and enrolled participants will be collected, shared, and maintained in order to protect confidentiality before, during, and after the trial	_____13______
Declaration of interests	28	Financial and other competing interests for principal investigators for the overall trial and each study site	_____14______
Access to data	29	Statement of who will have access to the final trial dataset, and disclosure of contractual agreements that limit such access for investigators	_____13______
Ancillary and post-trial care	30	Provisions, if any, for ancillary and post-trial care, and for compensation to those who suffer harm from trial participation	_____13______
Dissemination policy	31a	Plans for investigators and sponsor to communicate trial results to participants, healthcare professionals, the public, and other relevant groups (eg, via publication, reporting in results databases, or other data sharing arrangements), including any publication restrictions	_____2, 6, 13__
	31b	Authorship eligibility guidelines and any intended use of professional writers	____________
	31c	Plans, if any, for granting public access to the full protocol, participant-level dataset, and statistical code	_____13______
**Appendices**			
Informed consent materials	32	Model consent form and other related documentation given to participants and authorised surrogates	____________
Biological specimens	33	Plans for collection, laboratory evaluation, and storage of biological specimens for genetic or molecular analysis in the current trial and for future use in ancillary studies, if applicable	____________

*It is strongly recommended that this checklist be read in conjunction with the SPIRIT 2013 Explanation & Elaboration for important clarification on the items. Amendments to the protocol should be tracked and dated. The SPIRIT checklist is copyrighted by the SPIRIT Group under the Creative Commons "Attribution-NonCommercial-NoDerivs 3.0 Unported" license.

For peer review only - http://bmjopen.bmj.com/site/about/guidelines.xhtml

BMJ Open

A longitudinal observational study of home-based conversations for detecting early dementia: Protocol for the CUBOId TV task

Journal:	*BMJ Open*
Manuscript ID	bmjopen-2022-065033.R1
Article Type:	Protocol
Date Submitted by the Author:	03-Nov-2022
Complete List of Authors:	Kumpik, Daniel; University of Bristol, Department of Engineering Mathematics<br>Santos-Rodriguez, Raul; University of Bristol, Department of Engineering Mathematics<br>Selwood, James; Bristol Medical School; University of Bristol, Department of Population Health Sciences<br>Coulthard , Liz ; Bristol Medical School; University of Bristol, Department of Translational Health Sciences<br>Twomey, Niall; University of Bristol, Department of Electrical & Electronic Engineering<br>Craddock, Ian ; University of Bristol, Department of Electrical and Electronic Engineering<br>Ben-Shlomo, Yoav; University of Bristol, Department of Population Health Sciences
**Primary Subject Heading**:	Neurology
Secondary Subject Heading:	Health informatics, Diagnostics, Geriatric medicine, Research methods
Keywords:	Dementia < NEUROLOGY, Health informatics < BIOTECHNOLOGY & BIOINFORMATICS, GERIATRIC MEDICINE, Neurology < INTERNAL MEDICINE, Adult neurology < NEUROLOGY, Neuropathology < NEUROLOGY

SCHOLARONE™
Manuscripts

A longitudinal observational study of home-based conversations for detecting early dementia: Protocol for the CUBOId TV task

Authors

Daniel Paul Kumpik[1], Raul Santos-Rodriguez[1], James Selwood[2], Elizabeth Coulthard[3], Niall Twomey[4], Ian Craddock[4], Yoav Ben-Shlomo[2].

1. Department of Engineering Mathematics, University of Bristol, Bristol, UK

2. Population Health Sciences, Bristol Medical School, University of Bristol, Bristol, UK

3. Translational Health Sciences, Bristol Medical School, University of Bristol, Bristol, UK

4. Department of Electrical & Electronic Engineering, University of Bristol, Bristol, UK

Author for correspondence: Daniel Kumpik, Department of Engineering Mathematics, Ada Lovelace Building, Tankard's Close, University Walk, Bristol BS8 1TW; dan.kumpik@bristol.ac.uk

Word count: 4243

For peer review only

ABSTRACT

Introduction

Limitations in effective dementia therapies mean that early diagnosis and monitoring are critical for disease management, but current clinical tools are impractical and/or unreliable, and disregard short-term symptom variability. Behavioural biomarkers of cognitive decline, such as speech, sleep and activity patterns, can manifest prodromal pathological changes. They can be continuously measured at home with smart sensing technologies, and permit leveraging of interpersonal interactions for optimising diagnostic and prognostic performance. Here we describe the ContinUous behavioural Biomarkers Of cognitive Impairment (CUBOId) study, which explores the feasibility of multimodal data fusion for in-home monitoring of mild cognitive impairment (MCI) and early Alzheimer's disease (AD). The report focuses on a subset of CUBOId participants who perform a novel speech task, the "TV task", designed to track changes in ecologically valid conversations with disease progression.

Methods and Analysis

CUBOId is a longitudinal observational study. Participants have diagnoses of MCI or AD, and controls are their live-in partners with no such diagnosis. Multimodal activity data were passively acquired from wearables and in-home fixed sensors over timespans of 8 to 25 months. At 2 time points participants completed the TV task over 5 days by recording audio of their conversations as they watched a favourite TV programme, with further testing to be completed after removal of the sensor installations. Behavioural testing is supported by neuropsychological assessment for deriving ground truths on cognitive status. Deep learning will be used to generate fused multimodal activity-speech embeddings for optimisation of diagnostic and predictive performance from speech alone.

Ethics and dissemination

CUBOId was approved by an NHS Research Ethics Committee (Wales REC; ref: 18/WA/0158) and is sponsored by University of Bristol. It is supported by the National Institute for Health Research (NIHR) Clinical Research Network West of England. Results will be reported at conferences and in peer-reviewed scientific journals.

Keywords

dementia, mild cognitive impairment, Alzheimer's disease, behavioural biomarkers, activities of daily living, speech, smart sensing technologies, multimodal data fusion, deep learning

For peer review only

ARTICLE SUMMARY

Strengths and limitations

- We characterise diagnostic trajectories across a broad range of behavioural domains relevant to cognitive decline "in the wild"
- Patients' live-in partners are contextually matched controls, accounting for differences across participants' home environments and lifestyles
- Neuropsychological measurements form ground truths for behavioural biomarkers of cognitive decline
- Limited sample size and restricted demographics may confer limited generalisability of findings to other populations
- Behavioural and neuropsychological testing timelines disrupted and desynchronised due to COVID-19

INTRODUCTION

Alzheimer's disease (AD) is one of the most common neurological diseases, affecting an estimated 42 million people worldwide [1]. AD causes a progressive loss of memory, executive, visuospatial and linguistic functions, which disrupts patients' ability to engage in functional activities of daily living (ADL) [2]. Therapeutic options are limited, and clinical interventions are likely more effective in very early or prodromal stages of AD, highlighting the value of early detection [3]. However, abnormal brain pathology can manifest decades before cognitive symptoms [4] and by the time of diagnosis 50% of patients are beyond the early stages [5].

Mild cognitive impairment (MCI), often a prodromal phase of AD, is characterised by forgetfulness, mood changes, and navigation, decision-making and word-finding problems, which are not sufficiently severe to interfere with ADL [6]. Up to 50% of individuals diagnosed with MCI develop AD within 5 years [7], but some MCI cases remain stable and some recover over time [8], complicating prediction of an individual patient's disease progression. Clinical investigations such as lumbar puncture and brain imaging are expensive and impractical for large-scale deployment [9,10], so clinicians have traditionally relied on cognitive screening tests [11]. However, these tests may be limited in validity, specificity, inter-examiner consistency, disregard short-term symptom variability, and usually require stressful visits to clinic, which can bias results [12,13]. They therefore provide a contextually sparse and relatively insensitive measure of subtle early signs of cognitive decline

For peer review only

[4,13]. Whilst blood biomarkers are close to clinical implementation and could revolutionise detection of molecular AD pathology [14], these are positive early in the course of the illness, so do not mirror clinical decline, and can yield false positives.

These limitations have driven recent interest in passive technologies for longitudinal monitoring of behavioural dementia biomarkers in naturalistic settings [15–18]. Preclinical disruptions in activities such as speaking [19], environmental navigation [20], sleep [21] and walking [22] can betray distal biomarkers such as wandering, circadian disturbances, apathy and agitation [23], which may be useful both in diagnosing MCI and predicting which patients with MCI may go on to develop AD. These ecologically valid, everyday behaviours can be measured continuously and unobtrusively in the home, using networked fixed sensors, or portable sensors in wearables, smartphones and tablets [16]. Fusion across sensing modalities permits fine-grained contextual analysis of how engagement in different ADL covaries over days, weeks and months, and automated machine learning (ML) techniques can leverage intraindividual variability reflective of early cognitive changes [15,24]. These technologies have potential to help clinicians build personalised and holistic digital phenotypes for prediction of neurodegeneration [17,25]. However, they remain insufficiently mature for full-scale clinical deployment [15].

Speech is a particularly interesting dementia biomarker because linguistic competence whilst cognitively healthy is a known predictor for developing AD [26,27]. AD disrupts morphological, lexical, semantic, syntactic and structural features of language [28], and can even be detected in acoustic, temporal and other paralinguistic features [29–31]. Speech is simple to acquire using modern smart devices and therefore is an attractive target for longitudinal in-home monitoring of cognitive decline [32,33]. Advances in automated speech recognition (ASR), natural language processing (NLP) and other ML technologies have contributed to promising results in classifying AD from recorded speech [34], and recent work has also shown that linguistic and paralinguistic features can help detect MCI [35]. However, classification remains more challenging for MCI than AD, probably because MCI patients' cognitive profiles are heterogenous and not as far removed from those of normal ageing [29,36].

Studies leveraging speech to diagnose dementia often train ML models on databases of structured speech gleaned using narrative tasks such as picture description. These provide a rich impression of semantic content, idea density, syntactic complexity, speech fluency and paralinguistic parameters, and discourse fluency [37], which could be important features for detecting prodromal AD. However, other than occasional interventions from a clinical investigator (which are often discarded (e.g., [38]), narrative monologues are constrained by the stimulus material and impoverished in verbal

For peer review only - http://bmjopen.bmj.com/site/about/guidelines.xhtml

interactions found in natural conversations. Spontaneous conversations require executive attentional, metacognitive and linguistic dexterities not demanded by structured speech tasks that may be useful for detecting MCI due to AD or predicting cognitive decline [37,39]. Moreover, picture description tasks mostly preclude dyadic signs of cognitive decline, such as prompting in response to word-finding problems [40]. Indeed, the importance of dyadic features for dementia diagnosis is emphasised by the recent finding that it is possible to classify AD from picture description tasks with an accuracy of 78% from the speech of the interviewer alone [41]. Whilst there is acknowledgement that leveraging ecologically valid, naturalistic conversations for diagnosing dementia has merit [42,43], speech is rarely collected via passive, *unprompted* observation of patients and their partners “in the wild”, prompting recent calls for investigation of ecologically acquired, conversational speech relevant to ADL that could confer better diagnostic generalisability [44].

The ContinUous behavioural Biomarkers Of cognitive Impairment (CUBOId) study at the University of Bristol (UoB) is part of the EPSRC Sensor Platform for HEalthcare in a Residential Environment (SPHERE) Interdisciplinary Research Collaboration [45]. CUBOId aims to advance the state of the art in multimodal in-home sensing for novel dementia-related behavioural biomarkers [46] such as sleep disturbances, partner shadowing, wandering and disrupted conversational speech. We will develop novel computational algorithms to infer biomarkers of cognitive decline as measured using sensor networks in MCI and AD patients’ own homes, permitting autonomous, continuous, long-term behavioural sensing. Where possible these will be validated against neuroimaging (amyloid PET) data and neuropsychological progression over time. The patient’s live-in companions are also participants, to act as environmentally matched controls and shed light on interactional biomarkers of cognitive decline. Because the companion is familiar to the patient, we hope to record behavioural interactions that have high ecological validity.

Here we report on the data acquisition and analysis protocol for CUBOId’s novel conversational speech task, the “TV task”. We give an overview of CUBOId’s in-home sensing methods and outline how concurrent sensor readings from environmental sensors, cameras and wearable accelerometers can be combined, and further fused with conversational speech features, to improve diagnosis from speech alone. Our objectives are as follows:

1. To develop ML models capable of leveraging home-acquired, natural speech for automated prediction, diagnosis and longitudinal tracking of cognitive decline. For this we will establish ground truths on early speech biomarkers of MCI/AD from neurological and neuropsychological tests.

For peer review only - http://bmjopen.bmj.com/site/about/guidelines.xhtml

2. To investigate how temporal variability in linguistic proficiency is reflected in clinically relevant variability in ADL behaviours. For example, we hypothesise that periods of agitation, wandering and sleep disturbances will precede disturbed speech as revealed by acoustic and prosodic changes, hesitations, mnemonic search markers and endogenous (e.g., rapid speech) and exogenous (e.g., calming conversational interactions) linguistic features.

3. To use insights from Objective 2 and data fusion techniques to leverage our multi-modal data streams by deriving latent relational embeddings between speech and multiple sensor readouts for optimisation of diagnostic and predictive performance from speech alone.

Whilst CUBOId's sensing regime is intended to be comprehensive, we aim to define the minimum dataset required for disease detection and monitoring, to enable more cost-efficient future use with simpler data capture methods. A further outcome will be pilot data on effect sizes sufficient to generate a meaningful power calculation to guide future studies.

METHODS

This is a longitudinal observational "proof of concept" study to examine speech and other behavioural biomarkers amongst cases with MCI or early AD and their partners, who act as controls. Speech is repeatedly measured over time in parallel with repeat neuropsychology and continuous multimodal home sensing. This permits investigation of whether speech-based diagnosis and monitoring of early dementia can be optimised using simultaneously recorded behavioural data. CUBOId started on 22/03/2019 and runs until 31/12/2022.

Patient and public involvement (PPI)

Prior to CUBOId deployments, SPHERE ran co-design workshops and publicly trialled its consent processes. On average, 86% properly understood the risks and 92% consented to take part. Patients were involved in pilot experiments for CUBOId's behavioural tasks. A PPI advisory team gave recommendations on recruitment methods, materials provided to participants and other practical issues. We plan to involve the PPI team with dissemination of materials to participants, the public and healthcare professionals.

Participants and eligibility criteria

For peer review only - http://bmjopen.bmj.com/site/about/guidelines.xhtml

Although data collection is ongoing, recruitment has now ended. Participants were recruited via the cognitive neurology service at the North Bristol NHS Trust Brain Centre, the Bristol Dementia Wellbeing Service and the Research Institute for the Care of Older People in Bath. Informed consent was obtained by a clinical fellow (JS) at the North Bristol Trust Brain Centre.

Participant pairs, each consisting of an outpatient with a previous diagnosis of either MCI or AD (confirmed by JS & EC) and their partner or live-in carer with no such diagnosis, were eligible. Participants with MCI were offered MRI and amyloid PET brain scans on a voluntary basis to discern the likely aetiology of their cognitive complaint (e.g. AD, vascular disease, subjective cognitive disorder). Where available, these were complemented with routine cerebrospinal fluid measurements of amyloid-β 42 and total tau expressed as total tau/amyloid-β 42 ratio, using a validated cut-off used in clinical practice above which an AD diagnosis is thought likely.

Participants with severe comorbidities were not considered. Participants were selected according to the following criteria; (a) Full capacity to give informed consent; (b) At least 50 years old; (c) Competent English speaker; (d) Normal or corrected-to-normal vision; (e) Consent to have SPHERE installed in their home.

A conventional sample size calculation was not performed as CUBOId is a precursor to future larger scale evaluations if the results look hopeful. From the larger CUBOId participant pool 9 sets of participants are participating in the TV task, of which 8 are patient-companion pairs with one MCI or AD patient (4/8 female). All 8 participant pairs have one male and one female participant. An additional male MCI patient lives alone, but nevertheless verbally describes the TV show during each recording session, permitting insight into how TV task performance differs under monadic and dyadic conditions. The mean age (± SD) of MCI and AD patients at study entry was 71.4 ± 6.9 years, and the mean age of companions was 64 ± 14 years (this information was not available for one companion). Companions are usually the life partners of the patients (one case is an adult child who lives with their parent). 6/9 patients had prior MCI diagnoses and 3/9 had prior AD diagnoses.

Some participants have hearing problems, a known risk factor for cognitive decline. We will therefore issue participants with a custom hearing questionnaire derived from the Speech, Spatial and Qualities of Hearing questionnaire (short form) [47] and the Glasgow Hearing Aid Benefit Profile [48] (see supplementary material S1).

The novel paradigm of the TV task

For peer review only - http://bmjopen.bmj.com/site/about/guidelines.xhtml

This task was inspired by the UK TV show “Gogglebox”. Participants record their conversations as they watch a TV programme of their choice such as the news, soap operas or other regular shows. They do so for a minimum of 30 minutes, at roughly the same time of day, over 5 consecutive days. It is emphasised that these are idealised guidelines and that participants should prioritise daily activities and unforeseen events over the TV task recording schedule. Participants are therefore asked, at minimum, to provide 5 half-hour recordings regardless of spacing between recordings. To catch discrepancies in TV shows or timings of recordings across days, participants recite the date and time and the name of the show at the beginning of each recording.

Participants place the tablet between their normal sitting positions for watching TV, then activate the audio recording app and converse naturally whilst using the TV programme as a stimulus for conversation. This guideline is to emphasise our desire to acquire naturalistic conversation, but also to maximise the amount of speech we record. The nature of the conversation is largely determined by the TV show, with e.g., news, geographical culture shows, gameshows and soap operas stimulating conversations that are, respectively: topical, about places and holidays, participatory and collaborative, or commentaries on the story and characters. Participants are asked to leave the app recording during adverts and if possible, until the end of the show. Once participants acquire 5 half-hour recordings, they return the tablet to the CUBOId team via courier.

Speech recordings are made using a Samsung Galaxy SM-T580 tablet and a pre-installed app [1], chosen because it is free of advertisements and easy to use, whilst providing a good range of recording options. Audio files are recorded at a sample rate of 44.1 kHz in mono in WAV format, apart from one pilot data collection block, which was recorded in MP3 format.

Tablets were provided by the research team during home visits prior to COVID-19’s arrival, but are now couriered to participants. Couriered tablets, chargers and parcel tape for return packaging are disinfected with antiviral spray, and a minimum three-day period is enforced after packing before participants open the package. Then, at a pre-agreed time, participants are contacted by the CUBOId team and given training on the TV task either over the phone or securely using Zoom video conferencing software [49].

Participants are shown how to use the Audio Recorder app and how to delete a recording if necessary for privacy reasons. After this, companions and patients provide controlled speech recordings by reading aloud ten sentences from the Harvard list [50], a set of phonetically balanced

[1] https://github.com/Dimowner/AudioRecorder

For peer review only - http://bmjopen.bmj.com/site/about/guidelines.xhtml

English sentences used to assess speech intelligibility in telecommunications systems. These control recordings provide a template for training deep neural networks to diarise participants' speech.

Participants then perform the TV task at their convenience. Detailed instructions describing the recording procedure are included as a PDF file on the device home screen, and telephone support is available in case of unforeseen difficulties.

Impact of COVID-19 on TV task data collection timeline

COVID-19, and technician/researcher availability, have caused severe disruptions to CUBOId by desynchronising the behavioural tasks. We originally intended to collect behavioural data across 3-4 testing blocks per participant pair, held at roughly 0, 6 and 12 months after SPHERE installation, with the possibility of a fourth block at 18 months if possible. We intended to visit participants' homes to train them on the behavioural tasks, but we suspended SPHERE deployments in March 2020 and introduced remote testing protocols to mitigate risk to participants. One participant pair first performed the TV task prior to the pandemic and had a large gap (17 months) between the first and second TV task administrations. The remaining participant sets completed the first TV task block in January-March 2021, after which one set withdrew from the TV task. All remaining participant sets completed their second block in July-September 2021, six of which still concurrently had active SPHERE deployments installed. A third testing block will be completed before 31st December 2022, without sensors present in participants' homes. To date, 9 participant sets have completed TV task testing block 1, 8 have completed block 2, and block 3 is currently underway.

Neuropsychological and cognitive tasks

Participants completed baseline neuropsychological testing (administered by JS: see supplementary material S2) before SPHERE installation. We intended to conduct repeat assessments at 6 and 12 months follow-up, but COVID-19 restrictions delayed these until later in the study. The test battery was adapted from the recommendations of the European Prevention of Alzheimer's Dementia (EPAD) study [51]. Participants also completed the Alzheimer's Disease Assessment Scale – Cognitive subscale (ADAS-Cog), considered the gold standard for dementia research trials [52].

Participants' psychological wellbeing and sleep were assessed using the 6-Item State-Trait Anxiety Inventory [53], the Geriatric Depression Scale (Short Version) [54], the Epworth Sleepiness Scale [55] and the Pittsburgh Sleep Quality Index [56]. After COVID-19's arrival, neuropsychological and

For peer review only - http://bmjopen.bmj.com/site/about/guidelines.xhtml

cognitive assessments were conducted using the online video-conferencing platform Attend Anywhere [2].

To measure memory performance participants complete a memory task at home using the Mezurio smartphone app [57] after each TV task testing block. Participants are encouraged to play Mezurio at roughly the same time each day, for about 10 minutes per day, for 30 days.

Multimodal sensing with SPHERE

SPHERE sensor modalities are categorised as wearable, environmental or silhouette. Custom wrist-worn wearables record acceleration indicative of bodily activity, posture and movement, and room-level location via triangulation of received signal strength measurements at Bluetooth access points throughout the home. The environmental modality consists of room sensors for occupancy, temperature, humidity, water and electricity usage. Silhouette cameras yield whole-body pose as well as fine-grained location information whilst obfuscating identity to preserve privacy.

DATA ANALYSIS

We will use Python and associated libraries for all analyses unless otherwise stated. Features derived from SPHERE sensors and speech recordings will be selected by examining their contributions to the variance in each dataset, their correlations with clinical measures such as ADAS-Cog and from insights into how ADL and speech covary longitudinally with disease progression. After standardisation of data by the within-population standard deviation, effect sizes for power calculations in future studies will be estimated from differences between a) controls (partners) and patients; b) houses with MCI and AD patients; and c) baseline performance and follow-up testing or analysis (for progression over time) [58].

Location and activity detection from SPHERE sensor data

Dedicated pre-processing pipelines will be applied for different sensor types, but fusion across sensors will provide a spatially and temporally contextualised overview of ADL behaviours and anomalies. We will explore which sensors are best combined for different activities and at what level of integration, and also the optimal stages at which to fuse activity with location data, ranging from

[2] https://www.attendanywhere.com

For peer review only - http://bmjopen.bmj.com/site/about/guidelines.xhtml

early (naïve multimodal feature set integration) to late (hierarchical integration of posterior probabilities from deep learning models trained on individual feature sets) fusion strategies [24,59].

Activity detection and in-home localisation twill leverage the sequential nature of our data by recognizing that certain behavioural transitions follow others with characteristic probabilities (Figure 1). For example, people never transition directly from sitting to running, but often from sitting to standing- and then running. Similarly, changes in participant location can only proceed across adjacent positions in physical space. This sequential dependence of states can be modelled using Viterbi decoding, which tracks a maximum-likelihood path through a series of noisy, probabilistic transitions in a Hidden Markov Model (HMM) [59,60]. We will compare HMM activity classification and localisation performance to deep learning architectures capable of leveraging spatial and temporal relationships in the multimodal feature space such as convolutional neural networks and long short-term memory networks [24]. We will compare these with pipelines exploiting both activity and location data together for further disambiguation of behaviour.

At the highest analysis level, we will derive sensor feature combinations suggestive of periods of "abnormal" dementia-related behaviours. For example, wandering could be inferred from a joint comparison of the wearable's activity levels and location complexity [46].

Behaviours we have identified as likely to be indicative of cognitive decline include [2,46]:

- Wandering, and shadowing of the companion
- Sleep disturbances
- Agitation
- Apathy, sedentarisation

We will analyse these longitudinally to identify short periods of unusual behaviour and longer-term changes that may be indicative of disease progression- and anomalous events in the recent past or future that could be related to such changes, such as poor sleep during nights before an episode of agitation. To leverage these behaviours alongside our conversational speech data, we will use representation learning to generate novel latent activity/speech embeddings for dementia detection based on speech alone. We will project these novel features onto unseen speech samples after exploring the best subset of SPHERE sensors to combine with our paralinguistic and linguistic models, and also whether diagnostic performance is better at this low level of SPHERE sensor integration compared with, for example, combining speech with actograms derived from pre-integrated sensor data. Fusion techniques will include multiview and transfer learning.

For peer review only - http://bmjopen.bmj.com/site/about/guidelines.xhtml

Speech pre-processing

We will build deep learning pipelines comprising paralinguistic and NLP domains to detect MCI, and predict the probability of progression to AD, from conversational speech, after experimenting with low and high-level fusion strategies for paralinguistic and linguistic features. The speech recordings suffer from quality issues that will require a dedicated pre-processing and speaker diarisation pipeline. Firstly, the samples contain two participants who speak infrequently (and sometimes over each other) against a variable TV background. The TV produces structured sounds including music, speech and sound effects with unpredictable spectrotemporal statistics that often resemble the participants' voices (Figure 2). Our single-channel recording setup is challenging for standard source separation techniques, but because we have acquired controlled voice recordings from each participant, we will use a semi-supervised learning architecture (such as variational autoencoders) to derive latent features for target separation. PRAAT phonetics software [61] will be used for annotating participants' voices for evaluation purposes, and recordings will be transcribed for NLP modelling according to established protocols [62].

Acoustic and paralinguistic features

We will extract non-linguistic speech features at local (i.e., fast Fourier transform window duration), semi-local (i.e., central tendency and variability statistics of features calculated on time scales of seconds) and global (i.e., over an entire audio file) time scales. Guided by feature taxonomies in recent reviews [44,63], local features will include representations of the speech spectrogram, such as Mel-frequency cepstral coefficients, as well as spectral bandwidth, short-time energy and zero-crossing count. Semi-local features will comprise paralinguistic measures of vocal quality known to reflect neuropathology such as jitter and shimmer, and higher prosodic features such as intonation, stress and rhythm. At the global feature level, we will compute utterance statistics such as articulation rate, time spent talking and mean utterance duration, and temporal mnemonic features relating to hesitations. We will extract interactional and pragmatic features such as the number of turn changes and overlapping segments [64], number of repair sequences [65] and inter-turn silence duration [66].

Natural language processing

We will analyse transcripts of CUBOId participants' speech using NLP modelling and deep learning. Python's Natural Language Toolkit and similar open resources will be used to conduct analyses on cognition-relevant features at the lexical, connected language and semantic levels [28]. At the lexical level, tokenisation and stemming, morphological parsing and part-of-speech tagging will help derive

For peer review only - http://bmjopen.bmj.com/site/about/guidelines.xhtml

measures of lexical diversity. At the level of connected language, we will explore pragmatics, syntactic complexity and correctness, topic coherence, and sequence predictability measures of disordered thinking. At the semantic level we will characterise idea density, a measure of linguistic conceptual richness highly predictive of cognitive decline in later life, and we will experiment with word embeddings selected from pre-trained language models such as BERT [67], which have found a cutting-edge role in dementia detection studies.

ETHICS AND DISSEMINATION

A major issue for studies with vulnerable people is to ensure they understand and are willing participants. Therefore JS, a clinician with extensive experience of working with dementia patients, ensured that all participants had capacity to consent at the outset. Given CUBOId's longitudinal nature, it is possible that capacity may be lost over time. Should this occur the partner is asked their opinion as to whether or not it is appropriate to proceed based on the patient's previously stated preferences.

CUBOId is sponsored by UoB and was approved by the Wales Research Ethics Committee (REC) 7 (ref: 18/WA/0158) and the Health Research Authority (IRAS project ID 234027). CUBOId is supported by the National Institute for Health Research (NIHR) Clinical Research Network West of England. Protocol amendments and adverse events are referred to the REC and UoB. UoB has Public Liability and Professional Negligence insurance policies to cover the eventuality of harm to a research participant or University employee arising from design or management of the research. Unforeseen protocol deviations are reported to the Principal Investigator, and serious deviations to the Sponsor, immediately. If CUBOId is ended prematurely, the Principal Investigator will notify the UoB and Wales RECs, including the reasons for termination.

DATA STATEMENT

All individuals in the CUBOId team have unrestricted access to the full dataset. Results will be reported at conferences, in peer-reviewed scientific journals and on the UoB website.

Both the anonymised data and study documentation are stored on secure servers within UoB and shared according to UoB procedures and guidelines. Personally identifiable information is stored separately to research data. Data management procedures follow the stipulations of the General

For peer review only - http://bmjopen.bmj.com/site/about/guidelines.xhtml

Data Protection Regulation. In line with NIHR guidance, we obtained consent for participants' data to be stored for up to 20 years and shared anonymously with other researchers, after appropriate review to ensure it will be used according to the ethics approval.

Privacy issues relating to identification of participants from CUBOId video recordings are addressed by using cameras that retain only the silhouettes of participants before data transfer to SPHERE servers. Analogous concerns regarding TV task audio recordings are addressed through careful curation and highlighting of sensitive information at annotation time for future obfuscation of, e.g., names with aliases. If audio data are released in future the voices may be further obfuscated using neural anonymisation techniques.

SUMMARY

To our knowledge there is no full-scale deployment of in-home, real-world, longitudinal activity sensing technologies that is complemented by monitoring of naturalistic conversations and neuropsychological assessment of MCI or AD patients. By bringing together these behavioural domains, CUBOId will observe how they covary with disease progression, permitting construction of models that perform better than those trained on a single modality. Here, we present a novel protocol for recording speech from MCI and AD patients and their companions as they converse at home. We intend to develop, test and de-risk a novel speech-based diagnostic asset for the UK Dementias Research Institute community, which may be applied to cohorts in the Dementia Platform UK, and the international community.

Author contributions: YBS, EC, JS, RSR, NT & IC conceived and designed CUBOId. DK, YBS, EC, JS, RSR & NT designed the TV task, and DK collected the TV task data and drafted the manuscript. All authors reviewed the manuscript prior to submission.

Acknowledgements: We would like to thank Julia Carey, David Bailey and Danielle Hale for their support in administering CUBOId and in collecting data. We would also like to thank our participants for their involvement in CUBOId.

Competing interests: None declared.

Funding: This research was funded by an MRC Momentum award (grant MC/PC/16029), the EPSRC SPHERE Interdisciplinary Research Collaboration (grant EP/K031910/1) and the EPSRC Centre for Doctoral Training in Digital Health and Care, University of Bristol (grant EP/S023704/1).

For peer review only - http://bmjopen.bmj.com/site/about/guidelines.xhtml

For peer review only

Licence Statement

I, the Submitting Author has the right to grant and does grant on behalf of all authors of the Work (as defined in the below author licence), an exclusive licence and/or a non-exclusive licence for contributions from authors who are: i) UK Crown employees; ii) where BMJ has agreed a CC-BY licence shall apply, and/or iii) in accordance with the terms applicable for US Federal Government officers or employees acting as part of their official duties; on a worldwide, perpetual, irrevocable, royalty-free basis to BMJ Publishing Group Ltd ("BMJ") its licensees and where the relevant Journal is co-owned by BMJ to the co-owners of the Journal, to publish the Work in BMJ Open and any other BMJ products and to exploit all rights, as set out in our licence.

The Submitting Author accepts and understands that any supply made under these terms is made by BMJ to the Submitting Author unless you are acting as an employee on behalf of your employer or a postgraduate student of an affiliated institution which is paying any applicable article publishing charge ("APC") for Open Access articles. Where the Submitting Author wishes to make the Work available on an Open Access basis (and intends to pay the relevant APC), the terms of reuse of such Open Access shall be governed by a Creative Commons licence – details of these licences and which Creative Commons licence will apply to this Work are set out in our licence referred to above.

REFERENCES

1 Brookmeyer R, Johnson E, Ziegler-Graham K, et al. Forecasting the global burden of Alzheimer's disease. *Alzheimers Dement* 2007;**3**:186–91. doi:10.1016/J.JALZ.2007.04.381

2 Saari T, Hallikainen I, Hintsa T, et al. Neuropsychiatric symptoms and activities of daily living in Alzheimer's disease: ALSOVA 5-year follow-up study. *Int Psychogeriatr* 2020;**32**:741–51. doi:10.1017/S1041610219001571

3 Liberati G, Raffone A, Belardinelli MO. Cognitive reserve and its implications for rehabilitation and Alzheimer's disease. *Cogn Process* 2012;**13**:1–12. doi:10.1007/s10339-011-0410-3

4 Bateman RJ, Fagan AM, Holtzman DM, et al. Clinical and biomarker changes in dominantly inherited Alzheimer' s disease. *N Engl J Med* 2012;**367**:795–804. doi:10.1056/NEJMoa1202753

For peer review only - http://bmjopen.bmj.com/site/about/guidelines.xhtml

5 Second English National Memory Clinics Audit. London: Royal College of Psychiatrists, 2015. https://silo.tips/download/second-english-national-memory-clinics-audit-report

6 Langa KM, Levine DA. The Diagnosis and Management of Mild Cognitive Impairment: A Clinical Review. *JAMA* 2014;**312**:2551. doi:10.1001/JAMA.2014.13806

7 Petersen RC. Mild cognitive impairment as a diagnostic entity. *J Intern Med* 2004;**256**:183–94. doi:10.1111/J.1365-2796.2004.01388.X

8 Ganguli M, Dodge HH, Shen C, *et al.* Mild cognitive impairment, amnestic type: an epidemiologic study. *Neurology* 2004;**63**:115–21. doi:10.1212/01.WNL.0000132523.27540.81

9 Ruan Q, D'Onofrio G, Sancarlo D, et al. Potential neuroimaging biomarkers of pathologic brain changes in Mild Cognitive Impairment and Alzheimer's disease: A systematic review. *BMC Geriatr* 2016;**16**:1–9. doi:10.1186/S12877-016-0281-7

10 Mo JA, Lim JH, Sul AR, *et al.* Cerebrospinal Fluid β-Amyloid1–42 Levels in the Differential Diagnosis of Alzheimer's Disease—Systematic Review and Meta-Analysis. *PLoS One* 2015;**10**:e0116802. doi:10.1371/JOURNAL.PONE.0116802

11 Webster L, Groskreutz D, Grinbergs-Saull A, *et al.* Development of a core outcome set for disease modification trials in mild to moderate dementia: a systematic review, patient and public consultation and consensus recommendations. *Health Technol Assess* 2017;**21**. doi:10.3310/HTA21260

12 Tsoi KKF, Chan JYC, Hirai HW, *et al.* Cognitive Tests to Detect Dementia: A Systematic Review and Meta-analysis. *JAMA Intern Med* 2015;**175**:1450–8. doi:10.1001/JAMAINTERNMED.2015.2152

13 Brown J. The use and misuse of short cognitive tests in the diagnosis of dementia. *J Neurol Neurosurg Psychiatry* 2015;**86**:680–5. doi:10.1136/jnnp-2014-309086

14 Teunissen CE, Verberk IMW, Thijssen EH, *et al.* Blood-based biomarkers for Alzheimer's disease: towards clinical implementation. *Lancet Neurol* 2022;**21**:66–77. doi:10.1016/S1474-4422(21)00361-6

15 Piau A, Wild K, Mattek N, *et al.* Current state of digital biomarker technologies for real-life, home-based monitoring of cognitive function for mild cognitive impairment to mild Alzheimer disease and implications for clinical care: Systematic review. *J Med Internet Res*. 2019;**21**. doi:10.2196/12785

16 Gillani N, Arslan T. Intelligent Sensing Technologies for the Diagnosis, Monitoring and Therapy of Alzheimer's Disease: A Systematic Review. *Sensors* 2021;**21**:4249. doi:10.3390/S21124249

17 Palliya Guruge C, Oviatt S, Delir Haghighi P, *et al.* Advances in Multimodal Behavioral Analytics for Early Dementia Diagnosis: A Review. *Proceedings of the 2021 International Conference on Multimodal Interaction*;**13**. doi:10.1145/3462244

18 Alfalahi H, Khandoker AH, Chowdhury N, et al. Diagnostic accuracy of keystroke dynamics as digital biomarkers for fine motor decline in neuropsychiatric disorders: a systematic review and meta-analysis. *Sci Rep* 2022; **12**: 7690. https://doi.org/10.1038/s41598-022-11865-7

For peer review only - http://bmjopen.bmj.com/site/about/guidelines.xhtml

19 Drummond C, Coutinho G, Fonseca RP, et al. Deficits in narrative discourse elicited by visual stimuli are already present in patients with mild cognitive impairment. *Front Aging Neurosci* 2015;**7**:96. doi:10.3389/FNAGI.2015.00096

20 Howett D, Castegnaro A, Krzywicka K, *et al.* Differentiation of mild cognitive impairment using an entorhinal cortex-based test of virtual reality navigation. *Brain* 2019;**142**:1751–66. doi:10.1093/brain/awz116

21 Gagnon JF, Lafrenière A, Rauchs G, et al. Sleep in Normal Aging, Alzheimer's Disease, and Mild Cognitive Impairment. In: Nomikos G, Feltner D, eds. Handbook of Behavioural Neuroscience: Translational Medicine in CNS Drug Development. Elsevier, 2019:677–92. doi:10.1016/B978-0-12-813743-7.00045-1

22 Masse FAA, Ansai JH, Fiogbe E, et al. Progression of Gait Changes in Older Adults with Mild Cognitive Impairment: A Systematic Review. *J Geriatr Phys Ther* 2021;**44**:119–24. doi:10.1519/JPT.0000000000000281

23 Kales HC, Gitlin LN, Lyketsos CG. Assessment and management of behavioral and psychological symptoms of dementia. *BMJ* 2015;**350**. doi:10.1136/bmj.h369

24 Qiu S, Zhao H, Jiang N, et al. Multi-sensor information fusion based on machine learning for real applications in human activity recognition: State-of-the-art and research challenges. *Inf Fusion* 2022;**80**:241–65. doi:10.1016/J.INFFUS.2021.11.006

25 Kourtis LC, Regele OB, Wright JM, et al. Digital biomarkers for Alzheimer's disease: the mobile/wearable devices opportunity. *NPJ Digit Med* 2019;2:1–9. doi:10.1038/s41746-019-0084-2

26 Iacono D, Markesbery WR, Gross M, *et al.* The Nun Study: Clinically silent AD, neuronal hypertrophy, and linguistic skills in early life. *Neurology* 2009;**73**:665–73. doi:10.1212/WNL.0b013e3181b01077

27 Eyigoz E, Mathur S, Santamaria M, et al. Linguistic markers predict onset of Alzheimer's disease. *EClinicalMedicine* 2020;**28**:100583. doi:10.1016/j.eclinm.2020.100583

28 Clarke N, Foltz P, Garrard P. How to do things with (thousands of) words: Computational approaches to discourse analysis in Alzheimer's disease. *Cortex* 2020;**129**:446–63. doi:10.1016/J.CORTEX.2020.05.001

29 Gosztolya G, Vincze V, Tóth L, *et al.* Identifying Mild Cognitive Impairment and mild Alzheimer's disease based on spontaneous speech using ASR and linguistic features. *Comput Speech Lang* 2019;**53**:181–97. doi:10.1016/j.csl.2018.07.007

30 Haider F, de La Fuente S, Luz S. An Assessment of Paralinguistic Acoustic Features for Detection of Alzheimer's Dementia in Spontaneous Speech. *IEEE J Sel Top Signal Process* 2020;**14**:272–81. doi:10.1109/JSTSP.2019.2955022

31 Gonzalez-Moreira E, Torres-Boza D, Garcia-Zamora MA, et al. Prosodic speech analysis to identify mild cognitive impairment. *IFMBE Proc* 2015;**49**:580–3. doi:10.1007/978-3-319-13117-7_148

32 Fraser KC, Komeili M. Measuring Cognitive Status from Speech in a Smart Home Environment. *IEEE Instrum Meas Mag* 2021;**24**:13–21. doi:10.1109/MIM.2021.9513645

For peer review only - http://bmjopen.bmj.com/site/about/guidelines.xhtml

33 Liang X, Batsis JA, Zhu Y, *et al.* Evaluating voice-assistant commands for dementia detection. *Comput Speech Lang* 2022;**72**:101297. doi:10.1016/J.CSL.2021.101297

34 Petti U, Baker S, Korhonen A. A systematic literature review of automatic Alzheimer's disease detection from speech and language. *J Am Med Inform Assoc* 2020;**27**,1784–1797. doi:10.1093/jamia/ocaa174

35 Martínez-Nicolás I, Llorente TE, Martínez-Sánchez F, *et al.* Ten Years of Research on Automatic Voice and Speech Analysis of People With Alzheimer's Disease and Mild Cognitive Impairment: A Systematic Review Article. *Front Psychol* 2021;**12**:645. doi:10.3389/FPSYG.2021.620251/

36 König A, Satt A, Sorin A, et al. Automatic speech analysis for the assessment of patients with predementia and Alzheimer's disease. *Alzheimers Dement* 2015;**1**:112–24. doi:10.1016/j.dadm.2014.11.012

37 Mueller KD, Hermann B, Mecollari J, *et al.* Connected speech and language in mild cognitive impairment and Alzheimer's disease: A review of picture description tasks. *J Clin Exp Neuropsychol.* 2018;**40**:917–39. doi:10.1080/13803395.2018.1446513

38 Shah Z, Sawalha J, Tasnim M, *et al.* Learning Language and Acoustic Models for Identifying Alzheimer's Dementia From Speech. *Front Comput Sci* 2021;**0**:4. doi:10.3389/FCOMP.2021.624659

39 Asgari M, Kaye J, Dodge H. Predicting mild cognitive impairment from spontaneous spoken utterances. *Alzheimers Dement* 2017;**3**:219. doi:10.1016/J.TRCI.2017.01.006

40 Nasreen S, Hough J, Purver M. Rare-Class Dialogue Act Tagging for Alzheimer's Disease Diagnosis. In *Proceedings of the 22nd Annual Meeting of the Special Interest Group on Discourse and Dialogue* 2021;290-300. https://aclanthology.org/2021.sigdial-1.32

41 Pérez-Toro PA, Bayerl SP, Arias-Vergara T, et al. Influence of the Interviewer on the Automatic Assessment of Alzheimer's Disease in the Context of the ADReSSo Challenge. In *Proceedings of Interspeech 2021* 2021; 3785–3789. doi:10.21437/Interspeech.2021-1589

42 Gkoumas D, Wang B, Tsakalidis A, et al. A Longitudinal Multi-modal Dataset for Dementia Monitoring and Diagnosis. *arXiv* 2109.01537 [Preprint]. September 03, 2021 [cited 2022 Mar 28] https://doi.org/10.48550/arXiv.2109.01537

43 Davis BH, Pope C. Finding a balance: The Carolinas Conversation Collection. *Corpus Linguist Linguist Theory* 2011;**7**:143–61. doi:10.1515/CLLT.2011.007

44 de La Fuente Garcia S, Ritchie C, Luz S. Artificial Intelligence, speech and language processing approaches to monitoring Alzheimer's Disease: a systematic review. *J Alzheimers Dis* 2020;**78**:1547-1574.

45 Woznowski P, Burrows A, Diethe T, *et al.* SPHERE: A Sensor Platform for Healthcare in a Residential Environment. In: Designing, Developing, and Facilitating Smart Cities. Springer International Publishing 2017. 315–33. doi:10.1007/978-3-319-44924-1_14

46 Poyiadzi R, Yang W, Ben-Shlomo Y, et al. Detecting Signatures of Early-stage Dementia with Behavioural Models Derived from Sensor Data. *CEUR Workshop Proc* 2020;**2820**:23–7.

For peer review only - http://bmjopen.bmj.com/site/about/guidelines.xhtml

47 Moulin A, Vergne J, Gallego S, *et al.* A New Speech, Spatial, and Qualities of Hearing Scale Short-Form: Factor, Cluster, and Comparative Analyses. *Ear Hear* 2019;**40**:938–50. doi:10.1097/AUD.0000000000000675

48 Gatehouse, S. The Glasgow Hearing Aid Benefit Profile: What it measures and how to use it. *Hear J* 2000;**53**:10–18.

49 Zoom Video Communications Inc. Zoom Security White Paper. Retrieved 28 March 2022 from https://explore.zoom.us/docs/doc/Zoom-Security-White-Paper.pdf.

50 Williams CE, Hecker MHL. Relation between Intelligibility Scores for Four Test Methods and Three Types of Speech Distortion. *J Acoust Soc Am* 2005;**44**:1002. doi:10.1121/1.1911189

51 Ritchie K, Ropacki M, Albala B, et al. Recommended cognitive outcomes in preclinical Alzheimer's disease: Consensus statement from the European Prevention of Alzheimer's Dementia project. *Alzheimers Dement* 2017;**13**:186–95. doi:10.1016/J.JALZ.2016.07.154

52 Kueper JK, Speechley M, Montero-Odasso M. The Alzheimer's Disease Assessment Scale–Cognitive Subscale (ADAS-Cog): Modifications and Responsiveness in Pre-Dementia Populations. A Narrative Review. *J Alzheimers Dis* 2018;**63**:423–44. doi:10.3233/JAD-170991

53 Marteau TM, Bekker H. The development of a six-item short-form of the state scale of the Spielberger State—Trait Anxiety Inventory (STAI). *British J Clin Psychol* 1992;**31**:301–6. doi:10.1111/J.2044-8260.1992.TB00997.X

54 Sheikh JI, Yesavage JA. Geriatric Depression Scale (GDS): Recent evidence and development of a shorter version. *Clin Gerontol* 1986;**5**:165–73. doi:10.1300/J018V05N01_09

55 Johns MW. A New Method for Measuring Daytime Sleepiness: The Epworth Sleepiness Scale. *Sleep* 1991;**14**:540–5. doi:10.1093/SLEEP/14.6.540

56 Buysse DJ, Reynolds CF, Monk TH, *et al.* The Pittsburgh sleep quality index: A new instrument for psychiatric practice and research. *Psychiatry Res* 1989;**28**:193–213. doi:10.1016/0165-1781(89)90047-4

57 Lancaster C, Koychev I, Blane J, et al. Evaluating the Feasibility of Frequent Cognitive Assessment Using the Mezurio Smartphone App: Observational and Interview Study in Adults With Elevated Dementia Risk. *JMIR Mhealth Uhealth* 2020;**8**:e16142. doi:10.2196/16142

58 Cohen, J. (1988). Statistical Power Analysis for the Behavioral Sciences (2nd ed.). Routledge. https://doi.org/10.4324/9780203771587

59 Kozlowski M, Byrne D, Santos-Rodriguez R, *et al.* Data fusion for robust indoor localisation in digital health. *2018 IEEE Wireless Communications and Networking Conference Workshops, WCNCW 2018* 2018;302–7. doi:10.1109/WCNCW.2018.8369009

60 Lentzas A, Vrakas D. Non-intrusive human activity recognition and abnormal behavior detection on elderly people: a review. *Artif Intell Rev* 2020;**53**:1975–2021. doi:10.1007/S10462-019-09724-5

61 Boersma P, Weenink D. Praat: doing phonetics by computer [Computer program]. Version 6.2.10, retrieved 28 March 2022 from http://www.praat.org/.

For peer review only - http://bmjopen.bmj.com/site/about/guidelines.xhtml

62 Macwhinney B. Tools for Analyzing Talk Part 1: The CHAT Transcription Format. Carnegie, 2022. Retrieved 28 March 2022 from https://talkbank.org/manuals/CHAT.pdf. https://doi.org/10.21415/3mhn-0z89

63 Voleti R, Liss JM, Berisha V. A Review of Automated Speech and Language Features for Assessment of Cognitive and Thought Disorders. *IEEE J Sel Top Signal Process* 2020;**14**:282–98. doi:10.1109/JSTSP.2019.2952087

64 Young JA, Lind C, van Steenbrugge W. A conversation analytic study of patterns of overlapping talk in conversations between individuals with dementia and their frequent communication partners. *Int J Lang Commun Disord* 2016;**51**:745–56. doi:10.1111/1460-6984.12245

65 Samuelsson C, Hydén LC. Collaboration, trouble and repair in multiparty interactions involving couples with dementia or aphasia. *Int J Speech Lang Pathol* 2017;**19**:454–464. doi:10.1080/17549507.2016.1221448

66 Nasreen S, Rohanian M, Hough J, et al. Alzheimer's Dementia Recognition From Spontaneous Speech Using Disfluency and Interactional Features. *Front Comput Sci* 2021;**3**:1–15. doi:10.3389/fcomp.2021.640669

67 Roshanzamir A, Aghajan H, Soleymani Baghshah M. Transformer-based deep neural network language models for Alzheimer's disease risk assessment from targeted speech. *BMC Med Inform Decis Mak* 2021;**21**:1–14. doi:10.1186/S12911-021-01456-3

For peer review only

For peer review only - http://bmjopen.bmj.com/site/about/guidelines.xhtml

FIGURE LEGENDS

Figure 1. Hidden Markov Models for activity detection. Activity transitions can be modelled as state changes in a Markov chain, with hidden activity states x_{ti} at time *t* represented by observed sensor (e.g., wearable accelerometer) inputs z_{ti}. Activities are assumed to transition sequentially with fixed probabilities obtained empirically (shown by the thickness and shading of the grey arrows): in this example, walking generally transitions to standing more often than to sitting, and never directly to lying down or cycling because a standing transition is required first. Maximum likelihood estimation over the entire sequence of sensor inputs is used to obtain an optimal activity transition sequence.

Figure 2. TV task speech pre-processing for speaker diarisation. *Top*, raw speech waveform with overlapping speakers 1 and 2 labelled, as well as the TV. *Middle*, speech spectrogram with zero-crossing count, a feature useful for detecting voiced and unvoiced speech, overlaid. *Bottom*, silence removal using a naïve energy detector after careful denoising.

For peer review only

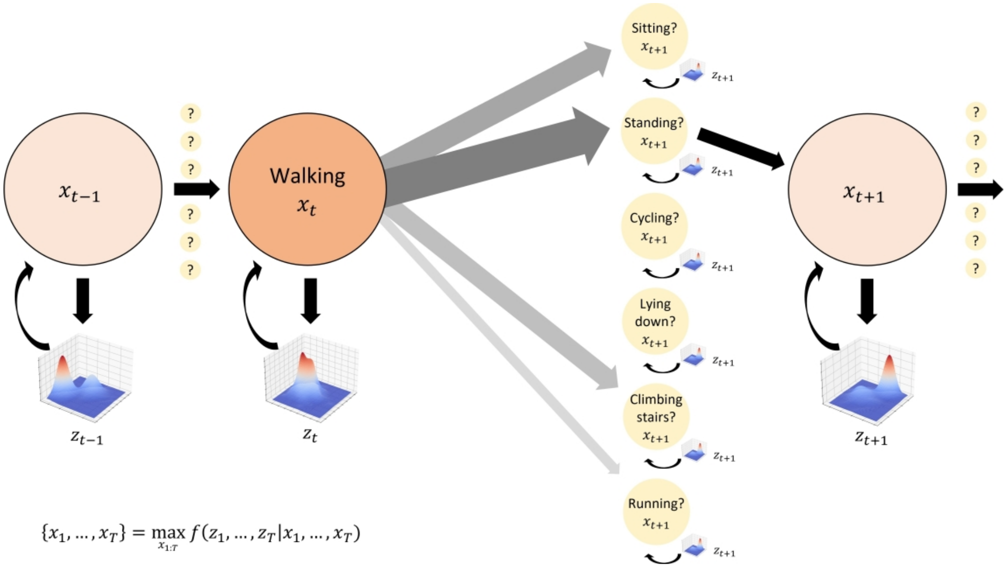

Figure 1. Hidden Markov Models for activity detection. Activity transitions can be modelled as state changes in a Markov chain, with hidden activity states x_ti at time t represented by observed sensor (e.g., wearable accelerometer) inputs z_ti. Activities are assumed to transition sequentially with fixed probabilities obtained empirically (shown by the thickness and shading of the grey arrows): in this example, walking generally transitions to standing more often than to sitting, and never directly to lying down or cycling because a standing transition is required first. Maximum likelihood estimation over the entire sequence of sensor inputs is used to obtain an optimal activity transition sequence.

91x51mm (1200 x 1200 DPI)

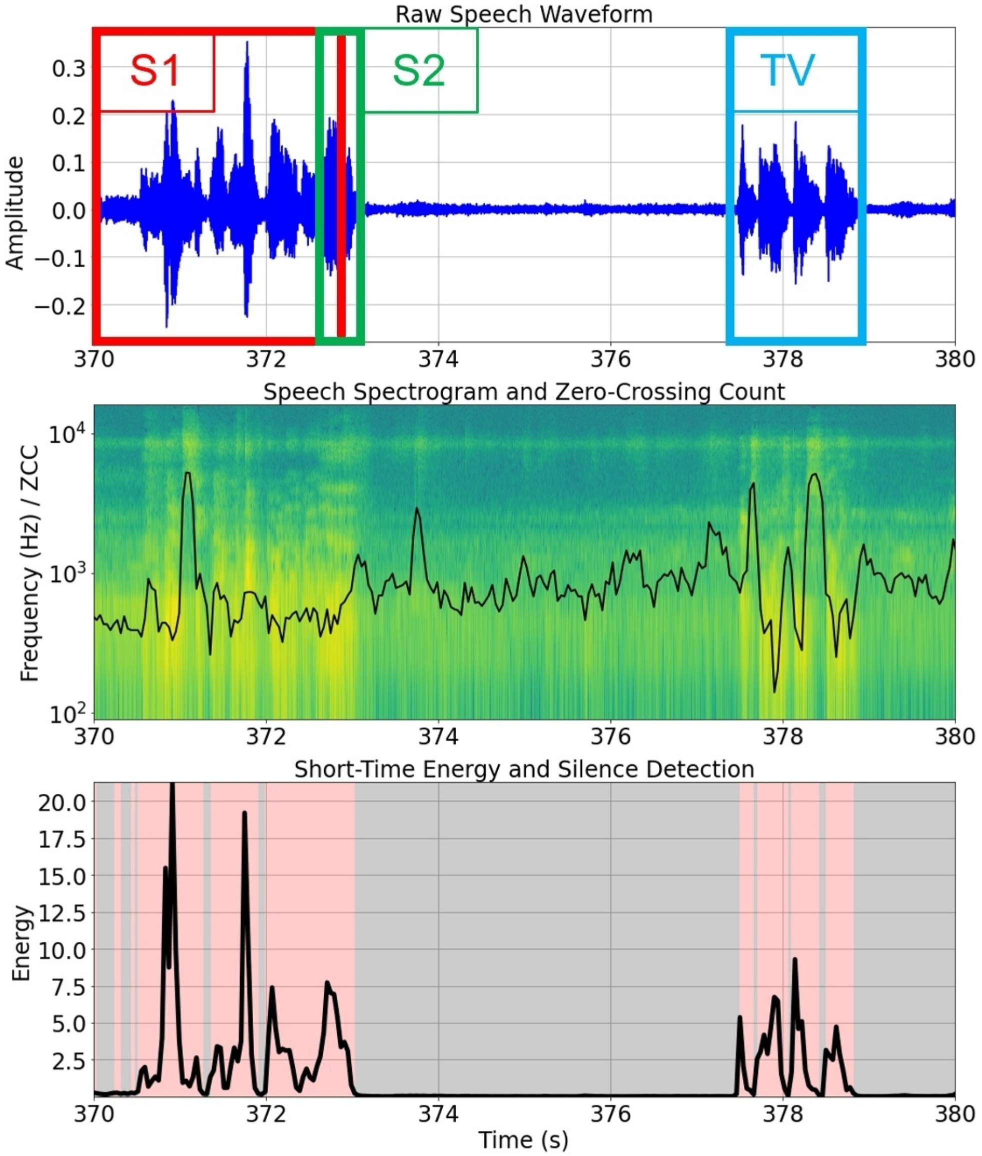

Figure 2. TV task speech pre-processing for speaker diarisation. Top, raw speech waveform with overlapping speakers 1 and 2 labelled, as well as the TV. Middle, speech spectrogram with zero-crossing count, a feature useful for detecting voiced and unvoiced speech, overlaid. Bottom, silence removal using a naïve energy detector after careful denoising.

96x112mm (300 x 300 DPI)

SUPPLEMENTARY MATERIAL

Supplementary Material S1 (overleaf). Hearing questionnaire to be issued to participants

For peer review only

CUBOId HEARING ASSESSMENT QUESTIONNAIRE

NAME: ______________________________

CURRENT AGE: ______________________________

TODAY'S DATE: ______________________________

I WILL BE COMPLETING THIS FORM (please tick only one box):

Myself ☐

With the help of my study partner ☐

Study partner completed form ☐

HEARING AID STATUS: I wear a hearing aid in my (please tick any that apply):

LEFT EAR ☐ RIGHT EAR ☐

We will ask you to answer 24 questions regarding your hearing experience and abilities. We would like both the CUBOId Main Participant and the Study Partner to answer these questions in relation to their own hearing. All questions require rating your hearing experience on a scale. The questions are in 2 sections, with instructions given at the beginning of each section. The first section asks questions about your "auditory quality of life", and the second section asks questions regarding whether using hearing aids provides any benefit for those difficulties when watching TV. The questions in each section are subtly different so please answer all questions in all sections if possible- if an answer does not apply to you (for example, you do not wear hearing aids), please answer "not applicable".

For peer review only

SECTION 1

15-item Speech, Spatial and Qualities of Hearing Questionnaire

These 15 questions inquire about aspects of your ability and experience while hearing and listening in different situations. For each question, put a mark, such as a cross, anywhere on the scale shown against each question. For example, putting a mark at 10 means that you would be perfectly able to do or experience what is described in the question. Putting a mark at 0 means you would be quite unable to do or experience what is described. As an example, question 1 asks about having a conversation with someone while the TV is on at the same time. If you are well able to do this, then put a mark toward the right-hand end of the scale. If you could follow about half the conversation in this situation put the mark around the mid-point, and so on. We expect that all the questions are relevant to your everyday experience, but if a question describes a situation that does not apply to you, put a cross in the “not applicable” box. Please also write a note next to that question explaining why it does not apply in your case.

1. **You are talking with one other person and there is a TV on in the same room. Without turning the TV down, can you follow what the person you’re talking to says?**

Not at all *Perfectly*

0 1 2 3 4 5 6 7 8 9 10 Not applicable ☐

2. **You are in a group of about 5 people in a busy restaurant. You CAN see everyone else in the group. Can you follow the conversation?**

Not at all *Perfectly*

0 1 2 3 4 5 6 7 8 9 10 Not applicable ☐

3. **You are talking with one other person. There is continuous background noise, such as a fan or running water. Can you follow what the person says?**

Not at all *Perfectly*

0 1 2 3 4 5 6 7 8 9 10 Not applicable ☐

4. You are in a group of about 5 people in a busy restaurant. You CANNOT see everyone else in the group. Can you follow the conversation?

Not at all ... *Perfectly*

0 1 2 3 4 5 6 7 8 9 10 Not applicable ☐

5. You are in conversation with one person in a room where there are many other people talking. Can you follow what the person you are talking to is saying?

Not at all ... *Perfectly*

0 1 2 3 4 5 6 7 8 9 10 Not applicable ☐

6. You are sitting around a table or at a meeting with several people. You can't see everyone. Can you tell where any person is as soon as they start speaking?

Not at all ... *Perfectly*

0 1 2 3 4 5 6 7 8 9 10 Not applicable ☐

7. You are outside. A dog barks loudly. Can you tell immediately where it is, without having to look?

Not at all ... *Perfectly*

0 1 2 3 4 5 6 7 8 9 10 Not applicable ☐

8. You are standing on the footpath of a busy street. Can you hear right away which direction a bus or truck is coming from before you see it?

Not at all ... *Perfectly*

0 1 2 3 4 5 6 7 8 9 10 Not applicable ☐

9. **Can you tell from the sound of their voice or footsteps which direction a person is moving, for example, from your left to your right, or right to left?**

Not at all *Perfectly*

0 1 2 3 4 5 6 7 8 9 10 Not applicable ☐

10. **Do you have the impression of sounds being exactly where you would expect them to be?**

Not at all *Perfectly*

0 1 2 3 4 5 6 7 8 9 10 Not applicable ☐

11. **Do you find it easy to recognise different people you know by the sound of each one's voice?**

Not at all *Perfectly*

0 1 2 3 4 5 6 7 8 9 10 Not applicable ☐

12. **Do you find it easy to distinguish different pieces of music that you are familiar with?**

Not at all *Perfectly*

0 1 2 3 4 5 6 7 8 9 10 Not applicable ☐

13. **Can you tell the difference between different sounds, for example, a car versus a bus; water boiling in a pot versus food cooking in a frying pan?**

Not at all *Perfectly*

0 1 2 3 4 5 6 7 8 9 10 Not applicable ☐

14. When you listen to music, does it sound clear and natural?

Not at all — *Perfectly*

0 1 2 3 4 5 6 7 8 9 10 — Not applicable ☐

15. Do everyday sounds that you can hear easily seem clear to you (not blurred)?

Not at all — *Perfectly*

0 1 2 3 4 5 6 7 8 9 10 — Not applicable ☐

SECTION 2

Glasgow Hearing Aid Benefit Profile

In this section we describe 3 different listening scenarios and ask the same three questions about each of them. Please read all three scenarios before answering them, as they target similar but slightly different listening situations. Please circle the answer that best applies to your listening experience. Please assume that the term "hearing aid(s)" applies whether you wear one or two hearing aid(s). If you do not use hearing aid(s), please only answer the first question in each section and "N/A" for the other two. It would be helpful if you could also write a note saying "no HAs", so we can be sure that is why the question wasn't answered.

1. You are listening to the TV with other family or friends when the volume is adjusted to suit other people:

a. What level of difficulty do you have in this situation without hearing aid(s)?

N/A None Slight Moderate Great Cannot manage

b. In this situation, how much do your hearing aid(s) help you?

For peer review only - http://bmjopen.bmj.com/site/about/guidelines.xhtml

N/A	Not at all	A little	Moderately	Quite a lot	A great deal

c. In this situation, with your hearing aid(s), how much difficulty do you now have?

N/A	None	A little	Moderate	Quite a lot	A great deal

2. You are following TV when watching alone when there is no background noise:

a. What level of difficulty do you have in this situation without hearing aid(s)?

N/A	None	Slight	Moderate	Great	Cannot manage

b. In this situation, how much do your hearing aid(s) help you?

N/A	Not at all	A little	Moderately	Quite a lot	A great deal

c. In this situation, with your hearing aid(s), how much difficulty do you now have?

N/A	None	A little	Moderate	Quite a lot	A great deal

3. You are listening to someone talking to you while at the same time listening to a newscaster on TV. You are trying to follow what both people are saying.

a. What level of difficulty do you have in this situation without hearing aid(s)?

N/A	None	Slight	Moderate	Great	Cannot manage

b. In this situation, how much do your hearing aid(s) help you?

N/A	Not at all	A little	Moderately	Quite a lot	A great deal

c. In this situation, with your hearing aid(s), how much difficulty do you now have?

N/A	None	A little	Moderate	Quite a lot	A great deal

For peer review only

Supplementary Material S2. Test battery recommended in the European Prevention of Alzheimer's Dementia (EPAD) study. Table adapted from [51].

**Cognitive Domain**	**Test**	**Platform**
Reaction time/information processing speed/conceptual shifting/selective attention	Coding	RBANS
Verbal episodic memory	List learning	RBANS
	Story memory	RBANS
Visuospatial analysis	Figure copy	RBANS
	Line orientation	RBANS
Language	Picture naming	RBANS
	Semantic fluency	RBANS
Working memory	Digit span	RBANS
	Dot counting	NIH examiner/toolbox
Allocentric space	Four mountains task	iPad app
Paired associate learning	Paired associate learning	NIH examiner/toolbox

For peer review only

SPIRIT

STANDARD PROTOCOL ITEMS: RECOMMENDATIONS FOR INTERVENTIONAL TRIALS

SPIRIT 2013 Checklist: Recommended items to address in a clinical trial protocol and related documents*

**Section/item**	**Item No**	**Description**	**Addressed on page number**
**Administrative information**			
Title	1	Descriptive title identifying the study design, population, interventions, and, if applicable, trial acronym	_______1_______
Trial registration	2a	Trial identifier and registry name. If not yet registered, name of intended registry	______________
	2b	All items from the World Health Organization Trial Registration Data Set	______________
Protocol version	3	Date and version identifier	page headers
Funding	4	Sources and types of financial, material, and other support	_______14_____
Roles and responsibilities	5a	Names, affiliations, and roles of protocol contributors	_______1, 14___
	5b	Name and contact information for the trial sponsor	_______13_____
	5c	Role of study sponsor and funders, if any, in study design; collection, management, analysis, and interpretation of data; writing of the report; and the decision to submit the report for publication, including whether they will have ultimate authority over any of these activities	______________
	5d	Composition, roles, and responsibilities of the coordinating centre, steering committee, endpoint adjudication committee, data management team, and other individuals or groups overseeing the trial, if applicable (see Item 21a for data monitoring committee)	______________

For peer review only

Introduction

Background and rationale	6a	Description of research question and justification for undertaking the trial, including summary of relevant studies (published and unpublished) examining benefits and harms for each intervention	_____3-5_____
	6b	Explanation for choice of comparators	_____4-5_____
Objectives	7	Specific objectives or hypotheses	_____5-6_____
Trial design	8	Description of trial design including type of trial (eg, parallel group, crossover, factorial, single group), allocation ratio, and framework (eg, superiority, equivalence, noninferiority, exploratory)	______6______

Methods: Participants, interventions, and outcomes

Study setting	9	Description of study settings (eg, community clinic, academic hospital) and list of countries where data will be collected. Reference to where list of study sites can be obtained	_____7-8_____
Eligibility criteria	10	Inclusion and exclusion criteria for participants. If applicable, eligibility criteria for study centres and individuals who will perform the interventions (eg, surgeons, psychotherapists)	______7______
Interventions	11a	Interventions for each group with sufficient detail to allow replication, including how and when they will be administered	_____7-9_____
	11b	Criteria for discontinuing or modifying allocated interventions for a given trial participant (eg, drug dose change in response to harms, participant request, or improving/worsening disease)	____12-13____
	11c	Strategies to improve adherence to intervention protocols, and any procedures for monitoring adherence (eg, drug tablet return, laboratory tests)	______8,9____
	11d	Relevant concomitant care and interventions that are permitted or prohibited during the trial	_____________
Outcomes	12	Primary, secondary, and other outcomes, including the specific measurement variable (eg, systolic blood pressure), analysis metric (eg, change from baseline, final value, time to event), method of aggregation (eg, median, proportion), and time point for each outcome. Explanation of the clinical relevance of chosen efficacy and harm outcomes is strongly recommended	______5______
Participant timeline	13	Time schedule of enrolment, interventions (including any run-ins and washouts), assessments, and visits for participants. A schematic diagram is highly recommended (see Figure)	______9______

For peer review only - http://bmjopen.bmj.com/site/about/guidelines.xhtml

Sample size	14	Estimated number of participants needed to achieve study objectives and how it was determined, including clinical and statistical assumptions supporting any sample size calculations	_____7_______
Recruitment	15	Strategies for achieving adequate participant enrolment to reach target sample size	_____________

Methods: Assignment of interventions (for controlled trials)

Allocation:

Sequence generation	16a	Method of generating the allocation sequence (eg, computer-generated random numbers), and list of any factors for stratification. To reduce predictability of a random sequence, details of any planned restriction (eg, blocking) should be provided in a separate document that is unavailable to those who enrol participants or assign interventions	_____________
Allocation concealment mechanism	16b	Mechanism of implementing the allocation sequence (eg, central telephone; sequentially numbered, opaque, sealed envelopes), describing any steps to conceal the sequence until interventions are assigned	_____________
Implementation	16c	Who will generate the allocation sequence, who will enrol participants, and who will assign participants to interventions	_____________
Blinding (masking)	17a	Who will be blinded after assignment to interventions (eg, trial participants, care providers, outcome assessors, data analysts), and how	_____________
	17b	If blinded, circumstances under which unblinding is permissible, and procedure for revealing a participant's allocated intervention during the trial	_____________

Methods: Data collection, management, and analysis

Data collection methods	18a	Plans for assessment and collection of outcome, baseline, and other trial data, including any related processes to promote data quality (eg, duplicate measurements, training of assessors) and a description of study instruments (eg, questionnaires, laboratory tests) along with their reliability and validity, if known. Reference to where data collection forms can be found, if not in the protocol	____7-10_____
	18b	Plans to promote participant retention and complete follow-up, including list of any outcome data to be collected for participants who discontinue or deviate from intervention protocols	______9______

Data management	19	Plans for data entry, coding, security, and storage, including any related processes to promote data quality (eg, double data entry; range checks for data values). Reference to where details of data management procedures can be found, if not in the protocol	______13_____
Statistical methods	20a	Statistical methods for analysing primary and secondary outcomes. Reference to where other details of the statistical analysis plan can be found, if not in the protocol	_____10-12____
	20b	Methods for any additional analyses (eg, subgroup and adjusted analyses)	______________
	20c	Definition of analysis population relating to protocol non-adherence (eg, as randomised analysis), and any statistical methods to handle missing data (eg, multiple imputation)	______________
**Methods: Monitoring**			
Data monitoring	21a	Composition of data monitoring committee (DMC); summary of its role and reporting structure; statement of whether it is independent from the sponsor and competing interests; and reference to where further details about its charter can be found, if not in the protocol. Alternatively, an explanation of why a DMC is not needed	______________
	21b	Description of any interim analyses and stopping guidelines, including who will have access to these interim results and make the final decision to terminate the trial	______________
Harms	22	Plans for collecting, assessing, reporting, and managing solicited and spontaneously reported adverse events and other unintended effects of trial interventions or trial conduct	_____13______
Auditing	23	Frequency and procedures for auditing trial conduct, if any, and whether the process will be independent from investigators and the sponsor	______________
**Ethics and dissemination**			
Research ethics approval	24	Plans for seeking research ethics committee/institutional review board (REC/IRB) approval	_____13______
Protocol amendments	25	Plans for communicating important protocol modifications (eg, changes to eligibility criteria, outcomes, analyses) to relevant parties (eg, investigators, REC/IRBs, trial participants, trial registries, journals, regulators)	_____13______

For peer review only - http://bmjopen.bmj.com/site/about/guidelines.xhtml

Consent or assent	26a	Who will obtain informed consent or assent from potential trial participants or authorised surrogates, and how (see Item 32)	_____12-13_____
	26b	Additional consent provisions for collection and use of participant data and biological specimens in ancillary studies, if applicable	______13______
Confidentiality	27	How personal information about potential and enrolled participants will be collected, shared, and maintained in order to protect confidentiality before, during, and after the trial	______13______
Declaration of interests	28	Financial and other competing interests for principal investigators for the overall trial and each study site	______14______
Access to data	29	Statement of who will have access to the final trial dataset, and disclosure of contractual agreements that limit such access for investigators	______13______
Ancillary and post-trial care	30	Provisions, if any, for ancillary and post-trial care, and for compensation to those who suffer harm from trial participation	______13______
Dissemination policy	31a	Plans for investigators and sponsor to communicate trial results to participants, healthcare professionals, the public, and other relevant groups (eg, via publication, reporting in results databases, or other data sharing arrangements), including any publication restrictions	______2, 6, 13__
	31b	Authorship eligibility guidelines and any intended use of professional writers	______________
	31c	Plans, if any, for granting public access to the full protocol, participant-level dataset, and statistical code	______13______
**Appendices**			
Informed consent materials	32	Model consent form and other related documentation given to participants and authorised surrogates	______________
Biological specimens	33	Plans for collection, laboratory evaluation, and storage of biological specimens for genetic or molecular analysis in the current trial and for future use in ancillary studies, if applicable	______________

*It is strongly recommended that this checklist be read in conjunction with the SPIRIT 2013 Explanation & Elaboration for important clarification on the items. Amendments to the protocol should be tracked and dated. The SPIRIT checklist is copyrighted by the SPIRIT Group under the Creative Commons "Attribution-NonCommercial-NoDerivs 3.0 Unported" license.
